# Authentication of Insect-Based Products in Food and Feed: A Benchmark Survey

**DOI:** 10.3390/insects16070729

**Published:** 2025-07-17

**Authors:** Aline Marien, Benjamin Dubois, Olivier Fumière, Abigaël Anselmo, Julien Maljean, Clémence Debailleul, Jean-François Morin, Frédéric Debode

**Affiliations:** 1 Quality and Authentication of Agricultural Products Unit, Knowledge and Valorization of Agricultural Products Department, Walloon Agricultural Research Center (CRA-W), 5030 Gembloux, Belgium; o.fumiere@cra.wallonie.be (O.F.); a.anselmo@cra.wallonie.be (A.A.); j.maljean@cra.wallonie.be (J.M.); c.debailleul@cra.wallonie.be (C.D.); 2 Bioengineering Unit, Life Sciences Department, Walloon Agricultural Research Center (CRA-W), 5030 Gembloux, Belgium; b.dubois@cra.wallonie.be (B.D.); f.debode@cra.wallonie.be (F.D.); 3 Eurofins Biologie Moléculaire France, Eurofins, 44323 Nantes, France; jeanfrancois.morin@ftfr.eurofins.com

**Keywords:** PCR, HTS, metabarcoding, insects, food, feed, authentication, regulations

## Abstract

The consumption and farming of insects are gaining global attention as sustainable alternatives to conventional protein sources. Industrial processing of insects into powders or pastes complicates species identification, raising concerns about product authenticity, food safety, and potential fraud. In Western countries, particularly in Europe, the sector is expanding under a stringent regulatory framework, especially regarding rearing substrates, which hinders economic development. This study aimed to assess the species authenticity of commercial insect-based food and feed products and detect the presence of animal-derived DNA from unauthorized substrates. A total of 119 samples (pure insect meals and processed products) were collected from various origins. Species-specific real-time PCR assays targeted *Tenebrio molitor*, *Hermetia illucens*, *Alphitobius diaperinus*, *Acheta domesticus*, *Bombyx mori*, and *Gryllodes sigillatus*, alongside assays for ruminant, porcine, and poultry DNA. High-throughput sequencing (HTS) using metabarcoding confirmed and broadened species detection. Most samples contained the declared species; however, cases of mislabeling, substitution, and cross-contamination were observed. A few insect meals contained animal DNA which could suggest potential use of prohibited substrates. These findings highlight the urgent need for standardized authentication methods and improved transparency to ensure regulatory compliance, consumer trust, and sustainable development of the insect-based sector.

## Simple Summary

Insects are increasingly used as ingredients in food and animal feed, but it is necessary to be sure that products contain only the insect species listed on their labels. This study tested 119 commercial products using two DNA-based methods, real-time PCR and metabarcoding, to check whether the insects claimed on the packaging were actually present. The results showed that 50% of the products contained insect species not listed on the label, or lacked the species that were declared. In particular, cross-contamination was observed when manufacturers worked with more than one type of insect, and some products even contained insects that are not currently allowed for use in the European Union. Some insect meals also contained traces of animal DNA, which may come from the substrates the insects were raised on. This could cause legal problems if these meals are used in certain types of animal feed. The study highlights the need for better quality control in the insect production chain. It also shows that DNA tests are useful tools for checking what insect species are really in a product. Ensuring transparency in insect-based foods and feeds is important to protect consumers, support fair trade, and build trust in this growing industry.

## 1. Introduction

For centuries, insects have been traditionally harvested in various parts of the world, such as Asia, Africa, and America. There are many species of edible insects around the world [1], and they are part of the diet for many ethnic groups [2]. In Europe, insect consumption is not yet a major part of dietary habits, but during the last fifteen years, insect farming has become increasingly interesting across the world, and particularly in the Western world [2,3]. Insect proteins are progressively considered to be a valuable alternative to traditional protein sources and are presented as a relevant tool to reduce the environmental impact of food and feed production [4]. Moreover, the nutritional value of insects is often emphasized for their high content of proteins and essential amino acids, healthy fats, fiber, vitamins, and minerals [5,6]. In addition, diverse applications in niche sectors like dietary supplements, sports nutrition, pharmaceuticals, cosmetics, and fertilizers allow for better valorization of insect products and by-products [7]. In fact, insects are very versatile and can be incorporated into foods as whole insects (boiled, fried or dried), or as whole insects processed into granular powder or paste [8]. Lastly, insects could play a major role in a circular economic system and the reduction in food waste thanks to their ability to grow on a wide variety of substrates [9].

Over time, industrial production units of insect proteins have emerged in developed and developing countries [10] but the largest potential for growth is expected in Europe [11] and North America [12,13]. Currently, in Europe, the largest market share for edible insects is represented by whole insects, followed by bars, snacks, specialty food ingredients, and pasta [8]. In Europe, insects and insect-based products used in foods fall under the Novel Food Regulation (EU) 2015/2283, indicating the specific insects that can be utilized and including “whole insects and their parts”. These products can be commercialized on the EU market only after a long process of up to 17 months, as described by Mancini et al. (2022) [14]. It includes the submission of an application to the European Commission, its communication to all the Member States, and the request for a scientific safety assessment by the European Food Safety Authority (EFSA). Based on this opinion, authorization is granted or not. At present, only certain products from four insect species have received approval to be placed on the European food market: frozen, dried, and powder forms of *Tenebrio molitor* (L.) larvae (yellow mealworm) [15,16], *Locusta migratoria* (L.) (migratory locust) [17], *Acheta domesticus* (L.) (house cricket) [18], and *Alphitobius diaperinus* (Panzer) larvae (lesser mealworm) [19], as well as partially defatted powder of *A. domesticus* (house cricket) [19]. For the labeling of novel foods containing insects intended for human consumption, Regulation (EU) No 1169/2011, also called the FIC (Food Information to Consumers) Regulation, is applicable. It stipulates that the food information provided to consumers, including the presence of ingredients derived from insects, must be clearly, accurately, and understandably identified on the label. Regarding the promotion of insect-based products to consumers, producers rely mainly on their websites and on events such as conferences, but it is important to note that these channels are restricted to regional markets [8]. For insects as feed, currently eight species are listed as allowed to be farmed: *Hermetia illucens* (L.) (black soldier fly), *Musca domestica* (L.) (common house fly), *T. molitor* (yellow mealworm), *A. diaperinus* (lesser mealworm), *A. domesticus* (house cricket), *Gryllodes sigillatus* (Walker) (banded cricket), *Gryllus assimilis* (F.) (field cricket) and *Bombyx mori* (L.) (silkworm) [20,21]. Live insects can be legally used as feed in the EU, except for ruminants, including for conventional food-producing animals like pigs, poultry, and aquaculture, provided they comply with general safety and marketing requirements. Processed animal proteins (PAPs) derived from insect species are considered animal by-products under Regulation (EC) No. 1069/2009 [22] and are permitted as feed, mainly for aquaculture, pigs, and poultry. Feeds containing insects are subject to Regulation (EC) No. 767/2009, which ensures the feed’s safety and hygiene, and must also comply with the general rules on feed hygiene outlined in Regulation (EC) 183/2005.

Despite this regulatory framework, the European insect sector is facing multiple legal restraints and challenges [7,9,14]. Presently, one of the main limitations of EU legislation is the restrictions on feed substrates, whatever the purpose (non-food, food, feed, as well as pet food). Regulation (EC) No 999/2001 [23] prohibits the use of PAPs in feed for farmed animals in order to prevent, control, and eradicate certain transmissible spongiform encephalopathies, such as bovine spongiform encephalopathy (BSE). Based on EFSA safety assessment studies, this total ban has been partially lifted for fish, pig, and poultry, but still remains for insects, limiting the rearing substrates to plant-based materials, while certain animal-based products such as milk and eggs are allowed [2,7]. This restriction continues to be a significant barrier to the development of the European insect industry, as it implies competition with the other livestock for the same feed materials [24]. Products consisting of or including meat and fish, such as waste from domestic use, restaurant, catering, former foodstuffs, and unsold products from supermarkets and industries, are banned, whereas they could positively promote the circular economy, maximize the potential of insects to mitigate the environmental footprint of conventional livestock, and boost the economic and environmental sustainability of insects farming [25]. These regulations are not uniform throughout the world. In North America, insects and insect-derived products are subject to a legislative framework [2,10]. As in Europe, insects considered as novel foods must be approved in Canada by the Canadian Food Inspection Agency and Health Canada, and in the United States (US) by the Food and Drug Administration (FDA) [10,26]. Under US law, food, and therefore insects, must be clean and wholesome (i.e., free from filth, pathogens, and toxins), must have been produced, packaged, stored, and transported under sanitary conditions, and must be appropriately labeled [10]. Nevertheless, some of these regulations are less restrictive than in Europe, particularly with regard to rearing substrates. In Canada, insects can be raised on a wide variety of substrates, including food and agricultural residues or industrial sludge [27]. In Central and South America, some countries, such as Mexico, Brazil, Colombia, and Argentina, have started to develop legislative frameworks for insect production and consumption [2]. In this context, in 2021, several insect species were approved in Brazil for use as food and feed [2]. In Argentina, new foods are officially incorporated into the Argentine Food Code, including a specific chapter on edible insects [2]. In Mexico, insect consumption is widespread, and all food products are regulated through mandatory standards called Normas Oficiales Mexicanas (NOMs) [10]. In 2021, Lähteenmäki-Uutela et al. [10] stated that edible insects did not yet have a specific NOM. However, there is a Mexican law on organic products which includes organic insect foods, lists the insect species concerned, specifies the conditions under which the insects must have been collected, and ensures that collection does not influence the ecosystem [10]. In Asia, legislation on insects varies from country to country. For example, in Thailand, the government has adopted a series of standards and regulations to regulate insect consumption and rearing, notably concerning cricket farming for export to the European market [2]. The standards for cricket breeding are called Good Agricultural Practices (GAP) and stipulate, for instance, that the farm must be protected from the weather, have a ventilation system, that the breeding equipment must be clean, dry, and free from impurities, that the food used as substrate must be of good quality, have been well packaged, and must not be rotten if vegetables, fruit, or plants are used [28]. In China, regulations on the production and sale of edible insects exist and are regulated by The China Food and Drug Administration (CFDA) [2] allowing some species to be recognized as food [29]. However, these regulations do not yet consider the safety aspect and therefore do not provide a legislative framework for insect rearing [29,30]. In Australia, regulations exist to control the import of insects for human and animal consumption, but there are no regulations governing the processing of insect-based products [2]. However, the pet food industry in Australia is self-regulated, and insect farmers tend to be transparent about their products [2]. Finally, in most African countries, there is no legal framework for insect rearing and consumption [2,31,32,33,34,35].

Moreover, the majority of insect-based products are subjected to several industrial treatments, such as different drying or nutrient extraction processes, and can be ground [36,37]. These multiple processes, designed to improve or maintain the nutritional quality and palatability [36], lead to the impossibility of visually or morphologically identifying the insects that compose them. This lack of ability to clearly authenticate insects, combined with non-standardized legislative frameworks around the world, can lead to shortcomings in terms of food and feed safety and to the implementation of fraudulent systems. As described by Fuso et al. (2024) [38], the different opportunities for fraud in the insect value chain can lead to safety issues such as allergenicity and microbiological risks, as well as chemical contamination (heavy metals, toxins, pesticides).

In this context, it is essential not only to have comprehensive legal frameworks at both national and international levels, but also to provide authentication techniques that are able to identify the insect species present in products available on the market, in order to guarantee food and feed safety, as well as to promote producers whose products comply with European regulations.

Among all the analytical methods available to check the conformity of products containing insects and to prevent fraud, DNA-based methods including conventional endpoint PCR, quantitative real-time PCR, and digital PCR remain reference methods, as they are well adapted to food and feed regulatory control. Most of the PCR methods already developed for the detection of insect species target mitochondrial markers located in cytochrome b (*cyt B*) [39], cytochrome oxidase I (*COI*) [40,41,42,43], NADH dehydrogenase [43] or *16S rDNA* dehydrogenase genes [41]. Although the fragments targeted are present in multiple copies per cell, increasing the sensitivity of the methods even with samples containing DNA degraded by an industrial process, this multicopy characteristic is highly variable and limits drastically any quantification purposes [44]. Nuclear genes present in a single-copy per cell, like the ones encoding *cadherin* [45,46,47], *wingless* [47] or *18S rDNA* [47] were also studied and used for the development of specific PCR tests.

More recently, high-throughput sequencing technologies using metabarcoding were also successfully applied [45,48]. DNA metabarcoding is a technique capable of providing more extensive results on the sample composition compared to a PCR test. DNA barcoding has been used successfully to detect and identify insect pests in samples submitted to a heat treatment at 118 °C for 18 min by using three short markers (less than 200 base pairs) located in *COI* and *16S rDNA* genes [49]. The metabarcoding approach was already used to distinguish over 1000 insect species [50] or to authenticate processed insect-based products [51]. However, the development of curated reference databases would further improve the effectiveness of the technique, as gaps, mislabeled entries, or simply the absence of a dedicated database may hamper the use of this approach [52].

Besides DNA-based methods, techniques targeting proteins are also under investigation to authenticate insect species in food and feed [53,54,55,56].

The present survey focused on insect meals and industrial multi-ingredient food and feed containing insect-derived ingredients, with the aim of conducting authentication tests using real-time PCR methods for the specific detection of *T. molitor* [47], *H. illucens* [44], *A. diaperinus* [46], *B. mori* [45], *A. domesticus* [39], and *G. sigillatus* [39]. To complement the PCR analysis, a DNA metabarcoding approach was also applied to provide additional insights into the composition and authenticity of the samples. In total, 119 samples were collected from both within and outside the European Union. In addition to species identification, insect flour (for food or feed) samples were analyzed using official PCR tests used for the detection of animal proteins in feed. Indeed, EU regulations prohibit the use of certain substrates (e.g., meat and bone meal, kitchen waste, manure, and animal excreta) in insect farming. Although some animal-derived products, such as egg-based and dairy products, are permitted, the presence of animal DNA—even from authorized sources—can raise regulatory concerns, as the current official methods based on DNA do not differentiate between allowed and prohibited DNA sources. This survey therefore aims to provide a comprehensive overview of the current market situation regarding the composition and compliance of insect-based products. It addresses key gaps in previous research by using of complementary methods, namely real-time PCR and DNA metabarcoding analyses, and by covering a wide diversity of insect species across samples collected both within and outside the EU. Moreover, it includes the detection of animal DNA from rearing substrates, a critical aspect not systematically explored in earlier studies.

## 2. Materials and Methods

### 2.1. Samples

One hundred and nineteen samples were collected from industries or purchased online. The samples were classified into four groups: 28 pure insect flours for food (Table 1), 27 multi-ingredient food products containing insects (Table 2), 21 pure insect meals for feed (Table 3), and 43 multi-ingredient feed products containing insects (Table 4). Table 1, Table 2, Table 3 and Table 4 compile information related to the samples while respecting confidentiality rules.

With regard to product origin, the country indicated refers to the origin of the final product, which does not necessarily mean that the insects it contains were reared in that location. Some companies display full transparency by specifying the origin of insect farming; for example, the brand 13 indicates that its products are made with cricket protein sourced from Thailand. In contrast, other companies provide no information about the provenance of the insects used.

The meal samples were placed in airtight containers and homogenized using a tumble mixer (REAX 20/12, Heidolph Scientific Products GmbH, Schwabach, Germany). Other samples were ground to 2 mm using a rotor mill (ZM200, Retsch, Haan, Germany), or with a knife mill equipped with single-use grinding bowls (Tube Mill control, IKA, Staufen, Germany) when the sample texture was not suitable for rotor milling; for example, cereal bars forming a paste-like consistency due to the presence of sugar and fruit.

### 2.2. DNA Extraction

Since the samples were intended to be analyzed using official PCR methods for the detection of animal proteins in livestock feed, the recommended extraction method for this type of analysis was used to obtain the DNA extracts. All samples (test portions of 100 mg) were then extracted following the method recommended by the European Union Reference Laboratory for Animal Proteins in feedingstuffs (EURL-AP) and based on the adaptation of the protocol of the ‘Wizard^®^ Magnetic DNA Purification System for Food’ kit (Promega, Madison, WI, USA). This method is described in the EURL-AP Standard Operating Procedure (SOP) [57]. The quantities tested for this purpose are also in line with the EURL-AP SOP.

All samples (test portions of 200 mg) were also extracted following the CTAB-based method described in Annex A.3.1 of the international standard ISO 21571:2005 [58] for metabarcoding analysis.

### 2.3. Real-Time PCR Method

All samples were analyzed with real-time PCR tests for *T. molitor* [47], *H. illucens* [44], *A. diaperinus* [46], *A. domesticus* [39], *B. mori* [45] and *G. sigillatus* (using the test from Daniso et al. [59] with a slight modification to the reverse primer described by Jilkova et al. [39]) detection. Table 5 shows the primers and probes used. The published PCR conditions were applied. Real-time PCR was performed on thermocycler LightCycler 480 (Roche Diagnostics Ltd., Rotkreuz, Switzerland) using the Brilliant II QPCR Low ROX Master Mix (Agilent Technologies, Santa Clara, CA, USA).

The single-species insect meal samples were analyzed using the three official PCR methods employed for the detection of processed animal proteins in animal feed. These three PCR methods target the detection of ruminant, pig, and poultry (chicken and turkey) DNA [60,61,62].

Cut-off values for ruminant, pig, and poultry detection were determined on a real-time PCR platform combining a LightCycler 480 thermocycler (Roche Diagnostics) with the Brilliant II QPCR Low ROX Master Mix (Agilent Technologies, Santa Clara, CA, USA), according to the protocols described by the EURL-AP [60,61,62]. The cut-off values were automatically generated using the Excel file provided for each PCR assay, available on the EURL-AP website (https://www.eurl.craw.eu/legal-sources-and-sops/method-of-reference-and-sops, accessed on 25 February 2025). A result for a reaction is considered positive when the Cq value obtained for that reaction is smaller than the cut-off value determined for the corresponding PCR test for the real-time PCR platform used.

All oligonucleotides were synthesized by Eurogentec (Seraing, Belgium). The PCR results obtained on a LightCycler 480 thermocycler (Roche Diagnostics Ltd.) were analyzed with LightCycler^®^ 480 Software (version 1.5.1.62).

### 2.4. Metabarcoding Approach

The insect composition of samples was also inferred using high-throughput sequencing in a metabarcoding assay. Each sample was analyzed with three different primer sets, in order to deduce a high-confidence taxonomic composition as a consensus from the results obtained with the different markers. These three markers targeted the *COI*, *12S*, and *28S rRNA* genes, respectively. This multi-marker metabarcoding approach was used to increase the accuracy of taxonomic composition assessments by mitigating the limitations of individual markers (such as PCR biases and gaps in reference databases), and it is expected to also reduce the incidence of ambiguous Orthopteran assignments, although perhaps not completely. Additionally, this strategy helped mitigate the issue of nuclear mitochondrial DNA segment (NUMT) detection (since sequencing of NUMTs can inflate diversity estimates or lead to species misassignments, especially for *COI*), particularly because the targeted *28S* marker is a nuclear locus.

The *COI* region was amplified using the primer set BF3 (5′- CCHGAYATRGCHTTYCCHCG-3′; [63]) + BR2 (5′- TCDGGRTGNCCRAARAAYCA-3′; [64]). The *12S rRNA* gene was amplified using the primers Dub12SF4 (5′- AAADAATTTGGCGGTRTTTTA-3′) and Dub12SR4 (5′- TAMNYCTACTWTGTTACGACTT-3′), while the primers Dub12SF3 (5′- AAACACGGACCAAGRAGTC-3′) and Dub12SR3a (5′- ACCCATTTAWAGTTTGAGAATAGGT-3′) were used to amplify the *28S rRNA* gene region. The PCRs were performed in triplicate. All 25 µL-PCR reactions were carried out using 5 µL of 5X GoTaq^®^ Flexi Buffer (Promega, Madison, WI, USA), 2.5 µL of 2mM dNTP mix (ThermoFisher Scientific, Waltham, MA, USA), 2.5 µL of 10 mM of bovine serum albumin (Roth, Newport Beach, CA, USA), 1.5 µL of 25 mM MgCl_2_ (Promega, Madison, WI, USA), 1.25 µL of 10 µM forward and reverse primers (Eurofins Genomics, Cologne, Germany), 0.15 µL of GoTaq^®^ G2 Flexi DNA Polymerase (Promega, Madison, WI, USA), 1 µL of DNA and 9.85 µL of nuclease-free water (QIAGEN, Hilden, Germany). Triplicate PCR products were pooled during purification using the NucleoSpin Gel and PCR Clean-up kit (Macherey-Nagel, Düren, Germany). The amplicon quality was checked using a Nanodrop One spectrophotometer (ThermoFisher Scientific, Waltham, MA, USA) and by running 5 µL of PCR products on a 1% agarose gel. The DNA concentration was measured using a Qubit 4 fluorometer (ThermoFisher, Waltham, MA, USA). For each sample, the three types of amplicons (*COI*, *12S*, and *28S*) were then pooled to obtain only one library per sample. This pooling step accounted for both amplicon concentration and length, ensuring that an equal number of each amplicon type was represented in the sequencing libraries. All libraries were sent to Eurofins Genomics (Cologne, Germany) for high-throughput sequencing on an Illumina MiSeq platform using 2 × 300 bp chemistry.

For bioinformatics analysis, raw sequencing data were imported into QIIME2 (2024.2 amplicon distribution). Demultiplexed paired-end reads were denoised using DADA2 (v1.26.0) [65] to generate amplicon sequence variants (ASVs). Taxonomy was assigned to the ASVs using the blastn standalone tool from the BLAST command line suite (v2.15.0) [66], using the following parameters: e-value = 0.001, identity percentage = 99%, and query coverage = 95%. In-house reference databases, developed and curated using the DB4Q2 pipeline [52], were used for this taxonomic analysis. They cover the entire Arthropoda phylum, except for the *COI* database, which was restricted to the Insecta class due to computational constraints. These databases contain 44,753 unique sequences for the *12S* database, 1,328,498 for the *COI* database, and 105,791 for the *28S* database. These sequences correspond, respectively, to 25,652, 358,681, and 62,702 species represented across the three databases. The reference databases used in this work (*COI*, *12S*- and *28S rRNA* genes), together with databases dedicated to other loci (*COII*, *CytB*, *16S* and *18S rRNA* genes), are freely available (https://doi.org/10.6084/m9.figshare.29313773). The TDmerger script (https://github.com/benn888/TDmerger, accessed on 8 July 2025) was used to infer consensus taxonomic composition from the results provided by the three PCR markers. The TDmerger script was run with the following parameters: “--remove-unassigned” set to “yes” (unassigned reads were discarded), “--min-abundance-threshold” set to “0.2” (taxa with a relative abundance lower than 0.2% were removed), and “--min-marker-threshold” set to “2” (taxa identified by fewer than 2 out of the 3 markers used were filtered out). These two types of thresholds were applied to minimize false positive results and artifacts as much as possible, although this approach carries a slight risk of excluding instances of low-level contamination.

## 3. Results

In our research, we focused on the procurement of “dry” insect samples, primarily in powdered, not whole insects, predominant formats on the market [67], where identification can be made visually, and then on products to be stored at room temperature, as these are easier to transport and store due to the absence of cold-chain requirements. This format is particularly suitable for experimental and analytical purposes. However, obtaining such samples has revealed a series of significant challenges.

One of the primary difficulties encountered in purchasing insect meal from the producer has been that these companies dedicated to the production of insect-based raw materials do not directly disclose the prices and quantities traded [67]. Furthermore, most commercial suppliers operate at a large scale and typically sell by the ton, often in bulk formats intended for feed production, refusing to sell small quantities. This makes it difficult for researchers to access the small quantities needed for scientific investigation.

Additionally, in several cases, we were only able to obtain samples after signing non-disclosure agreements (NDAs) with producers. These agreements restrict the dissemination of any commercially sensitive information, including brand names, product specifications, or manufacturer identity. As a result, all data related to these samples are presented in anonymized form to comply with confidentiality obligations.

Moreover, importation issues further complicated the acquisition of samples. In the European Union, the trading channels are not uniform, since regulation depends not only on the EU framework but also on a national basis in certain countries [67]. This is even more pronounced when sourcing from outside the European Union, where customs procedures can become a major bottleneck. Many producers, aware of the complexity and variability of international regulations on insect rearing and consumption, choose not to export their products, preferring to sell them only within their own national markets. These regulatory discrepancies often result in customs blocks or delays, making international procurement challenging.

Online sourcing has shown that some species are more readily available than others. Products derived from *T. molitor*, *A. domesticus*, and *A. diaperinus* are widely available and dominate online offerings for human consumption (Table 6). We found flours ready to be incorporated into culinary preparations, pasta, or even cereal-based products such as granola or energy bars. But cultural perceptions, including disgust and food neophobia, continue to limit the expansion of entomophagy in Western markets. In response, producers often develop insect-based products specifically designed to be more appealing to consumers, such as aperitif products, where the convivial nature of the occasion may encourage individuals to be more willing to try eating insects.

In the animal feed sector, accessibility to insect-derived ingredients also varies. The species *H. illucens* and *T. molitor* are the most widespread (Table 6) and are frequently found in pet food products. As to the *B. mori* species, it is mainly used in the feeding of ornamental fish.

While meals based on *A. diaperinus*, *G. sigillatus*, *G. assimilis*, and *M. domestica* are legally permitted, they are often difficult to source. In particular, *M. domestica* appears to be absent from the commercial feed market, likely due to rearing challenges and limited acceptance in pet or aquaculture contexts.

*T. molitor* is the most widely produced insect for food and feed globally, owing to its ease of rearing, minimal space requirements per unit of biomass, ability to survive solely on wheat bran or feed on various agricultural by-products [68], and its minimal water requirements for growth and development [69].

*A. domesticus* is also a species highly adapted to mass production and marketing thanks to its highly consistent life cycle. Its rearing can be readily organized to follow strict reproduction and harvesting schedules. The house cricket is among the most accepted insect species as human food [68].

As to *H. illucens*, it has rapidly become a flagship species in the animal feed insect production sector [70]. Due to its ability to digest a wide variety of organic wastes, its relatively short life cycle [71], its nutritional value as a protein- and fat-rich feed, and the production of a high-value soil amendment from its wastes [68].

These three species are tolerant to high larval density, which is not the case for *M. domestica*. A study by Kökdener and Kiper (2020) [72] showed that increasing larval density led to a significant reduction in pupal and adult weights, as well as decreased survival rates. Nutrient content also has an impact on life history parameters of *M. domestica*. The house fly also has a poor reputation because it is a notorious pest. Moreover, because house flies are a known vector of human and other vertebrate pathogens, depending on the diet selection, stricter precautions in breeding must be taken [68].

Whether in food or feed, in several cases, the exact insect species used in a product is not indicated. This is especially true for cricket-based items, which may only refer to “cricket” on the ingredient list without specifying the taxonomic identity.

### 3.1. Food: Pure Flour Samples

For food applications, 28 single-species insect flour samples were collected (samples 1 to 28). These included 10 samples of *T. molitor*, 7 of *A. domesticus*, 4 of *A. diaperinus*, 2 of *G. sigillatus*, 1 of *L. migratoria*, and 4 samples labeled simply as “crickets” without species-level identification.

Among the four *A. diaperinus* samples mentioned above (samples 18 to 21), one was labeled only as ‘*Alphitobius*’ or ‘buffalo larvae.’ While the term ‘buffalo larvae’ can refer to both species; *A. diaperinus* and *Alphitobius laevigatus* (F.) [46]. However, Marien et al. (2022) [46] reported that marketed *A. laevigatus* larvae are not readily available. Therefore, sample 19 was directly classified as containing *A. diaperinus*.

All 28 samples were analyzed with real-time species-specific targets and the results (Table 7) showed that insect flours from *T. molitor* (10 samples), *A. domesticus* (7 samples), and *A. diaperinus* (4 samples) were effectively detected when the corresponding species was labeled, with relatively early amplification signals (Cq values below 20 cycles for *A. domesticus* and *G. sigillatus*, and ranged between 20 and 27 cycles for *T. molitor* and *A. diaperinus*). Of the two *G. sigillatus* flours purchased (samples 22 and 23), one was correctly identified as such (sample 22), while the second (sample 23) was identified as *A. domesticus*. Indeed, the PCR signals obtained for this sample gave an average Cq value of 24 cycles, which is too early to be attributed to contamination and instead suggests a possible mislabeling or substitution since the supplier of this product also supplies *A. domesticus* flours. This result was further confirmed by HTS. Regarding the *L. migratoria* flour, it could not be tested by PCR for the expected species, but the HTS result showed that it was indeed *L. migratoria* flour. As noted by Jilkova et al. (2024) [39], products labeled simply as “cricket” without specifying the species are in fact typically based on *A. domesticus*. This was confirmed for the four cricket flours (samples 25 to 28) intended for food use that were analyzed in this study.

Concerning the purity level of insect flours specifically processed for food applications, it was observed that when a producer manufactures products from multiple insect species, cross-contamination between products can occur. This pattern was evident in products from brands 5, 6 and 8. Contamination is generally not problematic if the contamination remains minimal and involves species that are also authorized for human consumption. Such cases were observed in the following samples: *T. molitor* flours, samples 6 to 10, showed traces of *A. diaperinus* and/or *A. domesticus*; *A. domesticus* flours, samples 14 to 16, contained traces of *T. molitor*; and *L. migratoria* flour, sample 24 showed contamination with both *T. molitor* and *A. diaperinus*. Based on the Cq values obtained, the presence of *A. domesticus* in samples 24 and 20 (average Cq = 31 cycles), as well as *T. molitor* in sample 20 (average Cq = 30 cycles), suggests that these are no longer cases of incidental contamination but rather indicate a substantial presence of these species. As the species involved are authorized for human consumption, this does not pose a safety problem but it is nevertheless in contradiction with the FIC regulation. By contrast, the presence of non-authorized species may raise questions from a regulatory standpoint. This was the case for four samples—samples 6, 16, 17 and 21—in which *H. illucens* was detected. For sample 21, the level of contamination appeared to be very low, as indicated by the high Cq value, especially considering that the PCR assay used for *H. illucens* targets a multicopy gene. In the case of sample 6, which also revealed traces of *A. diaperinus*, the producer manufactures both *A. diaperinus* for food and *H. illucens* meals for feed, which could explain the observed cross-contamination. As for sample 17, the producer specifies that the insect protein used in their products originates from Thailand, but the species reared by their supplier are unknown; it is possible that *H. illucens* is also being produced.

Whether using PCR or HTS, the most abundant species detected is consistently the same across both analytical methods. However, differences can be observed in the detection of contaminant species. These discrepancies may result from the fact that DNA extraction methods differed between the two analytical approaches. Since the samples were also intended to be analyzed using official PCR methods for the detection of animal proteins in livestock feed, the method recommended by EURL-AP [57] was used to obtain the DNA extracts. In contrast, DNA extracts used for HTS were prepared using the CTAB method, which allows accurate quantification of DNA. Such quantification is not feasible with the ‘Wizard^®^ Magnetic DNA Purification System for Food’ kit due to the presence of residual magnetic beads used during extraction, which interfere with DNA measurement.

In addition to differences in extraction protocols, other factors such as method sensitivity may contribute to the observed discrepancies. On the one hand, HTS enables the detection of a broader range of species compared to PCR, since PCR is a targeted approach. On the other hand, HTS is generally less sensitive, as it involves fewer amplification cycles in order to avoid the formation of chimeric sequences and maintain, as much as possible, the representativeness of species diversity. Increasing the number of PCR cycles in HTS would lead to the overrepresentation of the most abundant species, thereby reducing the detectability of less abundant ones. Divergences between the two methods are particularly marked when PCR targets multi-copy genes—such as those used for detecting *H. illucens* and *A. domesticus*—which enhance sensitivity by amplifying multiple copies of the target sequence.

This pattern is further supported by the comparison of Cq values between samples. For single-copy targets, contaminations that remain undetected by HTS generally correspond to high Cq values in PCR. For example, sample 14, which yields a Cq of 38 cycles with the *T. molitor* PCR target, is not detected by HTS, whereas sample 20, with a Cq of 30 cycles for the same target, is successfully identified. In contrast, for PCR assays targeting multi-copy genes, a lower Cq values—expected due to the nature of the target— is not detected by HTS. For instance, sample 6, which tests positive for *H. illucens* with a Cq of 32 cycles, does not appear in the HTS results.

Using the metabarcoding approach (HTS), for sample 12, a very low proportion of reads (0.9%) were assigned to *Dermestes ater* (DeGeer), which may reflect trace contamination. This coleopteran species is known to be a pest of various food and non-food items worldwide [73].

Although these 28 single-species insect flour samples were intended for human consumption, they were also analyzed using official PCR tests developed for the detection of animal proteins in feed (Table 8). Indeed, whether the intended use is food or feed, in the European Union, farmed insects cannot be reared on substrates containing meat or bone, kitchen waste, or manure and animal excreta. However, certain animal-derived products (e.g., egg-based products, dairy products, non-ruminant blood products, etc.) are permitted.

The use of substrates containing animal-derived DNA, even when permitted, may raise regulatory concerns, as the current official PCR method does not distinguish between DNA from authorized and unauthorized sources. For example, the detection of ruminant DNA due to the use of milk-based substrates could raise suspicion of illegal use of bovine meat waste, or could also create problems for a feed manufacturer who incorporates this ingredient into production. This could be problematic, for instance, in the context of poultry feed production that also includes porcine PAPs. The detection of ruminant DNA could raise suspicion that it originates from ruminant PAPs, which are strictly prohibited in livestock feed due to the risk of bovine spongiform encephalopathy (BSE).

To assess whether products currently on the market may lead to such suspicions, the 28 samples were tested using the three official PCR assays.

No ruminant, porcine, or poultry DNA was detected in 20 out of the 28 samples. In 8 samples (samples 11–13, 15, 17, 26–28), ruminant DNA was detected, and one of these (sample 13) also contained porcine DNA. Among these 8 positive samples, 5 originated from countries outside the European Union (1 from the United Kingdom, 1 from New Zealand, and 3 from the United States (including 2 of the same brand)). These 5 samples also showed the earliest amplification signals, indicating higher DNA concentrations. For the samples from the United Kingdom and New Zealand, the manufacturers state, respectively, that the products are made with cricket protein sourced from Thailand and that the insects are sourced from sustainable, ethical, and reputable farms throughout Asia. The sample from New Zealand was the only one in which porcine DNA was also detected. It is also noteworthy that all positive results were obtained from flours derived from *A. domesticus*. Flours produced from other cricket species (*G. sigillatus* and *L. migratoria*), as well as from *T. molitor* and *A. diaperinus*, showed no detectable ruminant, porcine, or poultry DNA. These 17 samples all originated from the European Union.

### 3.2. Food: Multi-Ingredient Samples

Twenty-seven multi-ingredient foods (Table 9), from 11 different brands, labeled to contain insects, were purchased (samples 29 to 55). These included 7 samples of *T. molitor*, 11 of *A. domesticus*, 6 of *A. diaperinus* (including one labeled only as Buffalo worm), and 3 samples labeled simply as “crickets” without species-level identification. As with sample no. 19 among the single-species flours, and for the same reasons, sample no. 52, labeled as ‘buffalo worm’, was directly classified as containing *A. diaperinus*.

Real-time PCR targeting a species-specific DNA fragment confirmed the presence of *T. molitor*, *A. domesticus*, and *A. diaperinus* in samples where these species were labeled, except for sample 52 where no insect DNA could be amplified. To ensure that this negative result is not due to the presence of inhibitors, additional dilutions (20 and 40-fold dilutions) were analyzed. The sugar and salt content of this product may interfere with DNA extraction. However, this issue was observed in only one out of the 119 samples tested. For this product, HTS analysis confirmed that *A. diaperinus* is indeed the species present. The difference in results between the two methods may be due to the fact that the real-time PCR analyses were performed on DNA extracts obtained using the ‘Wizard Magnetic DNA Purification System for Food’ kit, whereas the HTS analyses were carried out on DNA extracted using the CTAB method.

As observed with the single-species flours, products labeled simply as ‘cricket’ were found to contain *A. domesticus*. This was confirmed in all three samples (53 to 55) analyzed in this category.

These results also indicate cross-contamination occurring when a manufacturer produces items containing different insect species. This pattern was particularly evident in the pasta products from brand 19. For instance, *T. molitor* DNA was detected in sample 53 pasta, which was based on *A. domesticus*, while *A. domesticus* DNA was found in sample 34, pasta made with *T. molitor*. A similar situation was observed with products from brand 1, where *A. diaperinus* was correctly detected as labeled, but unexpected traces of *A. domesticus* were also found. Notably, whole *A. domesticus* insects marketed under brand 1 are also available on the market. All of these cases of cross-contamination are not considered problematic, as they involve species that are also authorized for human consumption. However, they do raise questions about labeling accuracy. Including a statement indicating the possible presence of other insect species—similar to allergen warnings—could be considered, even though the allergenic risk is already addressed by the presence of insects in the product. In this context, the aim would not be to warn about allergenicity per se, but rather to ensure transparency regarding the actual insect composition.

The metabarcoding approach (HTS) provided additional information, revealing that products labeled as containing *A. domesticus* also contained *G. assimilis* (samples 43 to 45), with relative abundances of up to 31.8% in sample 43. Sample 45 also contained *Gryllus bimaculatus* (De Geer), with a relative abundance of 4.5%. Furthermore, one of the three samples labeled ‘cricket’ (sample 55) also contained *G. assimilis* (13.0% of the reads). The latter is a cricket species, just like *G. bimaculatus* found in sample 45, but neither of them is authorized for food use in the European Union. However, *G. assimilis* is an insect species authorized for use in animal feed in the European Union, whereas *G. bimaculatus* is farmed in Asia [74].

In sample 51, 2.6% of the reads were assigned to *Callosobruchus maculatus* (L.), a coleopteran species that is a prolific pest of stored leguminous products [75]. Contamination is therefore plausible, as the product’s composition indicates the presence of pea protein.

Regarding the detection of *H. illucens* by PCR, a species also not authorized for human consumption in the EU, its presence was less frequently observed in multi-ingredient food products (1 positive out of 27) compared to pure insect flour samples (4 positives out of 28). This is consistent with the low levels of *H. illucens* contamination observed in pure flours; once incorporated into a composite food matrix, this contamination is diluted and thus becomes undetectable.

### 3.3. Feed: Pure Meal Samples

For feed, 21 single-species insect meal samples were collected (samples 56 to 76). These included 9 samples of *H. illucens*, 5 of *T. molitor*, 2 of *B. mori*, 2 of *G. assimilis*, and 1 sample labeled simply as “crickets” without species-level identification. It was also possible to obtain two more non-conventional species through brand 5, namely *Blaptica dubia* (Serville) and *Gromphadorhina portentosa* (Schaum), which are reportedly intended for use in pet food. The availability of such diverse species is particularly valuable for research purposes.

Like previously, these samples were analyzed using real-time PCR with species-specific primers and an untargeted HTS approach. The results (Table 10) showed that insect meals from *H. illucens*, *T. molitor*, and *B. mori* are reliably detected by both methods when these species are declared (except for sample 67, for which HTS results were not received). Early Cq values were obtained by PCR (Cq values below 21 cycles for *H. illucens*, and ranging between 22 and 28 cycles for *T. molitor* and *B. mori*), except for sample 67, labeled as *T. molitor*, which yielded a late amplification signal (average Cq of 37 cycles), suggesting either significant DNA degradation due to processing or the presence of other components in addition to *T. molitor* meal. Notably, traces of *H. illucens* DNA were also detected in this sample. For sample 63, the reported Cq values correspond to the 10-fold diluted extracts. Although slight inhibition was observed in the undiluted extracts, it did not prevent amplification; however, the quality of amplification was improved with the 10-fold dilution.

Among the 17 samples derived from species authorized for use in animal feed and detectable with the PCR assays applied in this study, 10 (samples 56 to 63, 65 and 66) were found to contain only the expected species based on the PCR results. In the remaining 7 samples, at least one additional species was detected, but always with considerably later Cq values than those observed for the expected species. *A. diaperinus* and *H. illucens* DNA were also detected in a *T. molitor* meal (sample 68, brand 31) from Poland. Additionally, a *T. molitor* meal marketed for ornamental bird feed (sample 69, brand 32) contained traces of *A. domesticus* DNA, while the *H. illucens* meal from brand 5 (sample 64) contained traces of *T. molitor* DNA. The Cq values obtained for these two latter samples were very late, indicating a low quantity of DNA, which may explain why these results could not be confirmed by HTS. Both *B. mori* meal samples (samples 70 and 71) also contained *H. illucens* DNA. For these samples, HTS further revealed the presence of uncommon species such as *Tribolium castaneum* (Herbst), *Necrobia rufipes* (F.), and *Chrysomya megacephala* (F.).

Unlike all other cricket-labeled samples—which were consistently identified as *A. domesticus*—the cricket meal originating from Canada was identified by both real-time PCR and HTS as *G. sigillatus*. In this sample, *A. diaperinus* DNA was also detected by PCR, though with a substantially higher Cq value (35 cycles) compared to *G. sigillatus* (Cq = 21 cycles), suggesting a minor contamination.

Regarding the *G. assimilis*, *B. dubia*, and *G. portentosa* meals, PCR analysis could not be performed due to the absence of species-specific assays. However, HTS confirmed that the meals corresponded to the expected species. For the *B. dubia* meal (sample 75), *T. molitor* DNA was detected by real-time PCR with a mean Cq of 31 cycles, and this result was confirmed by HTS. Additionally, *H. illucens* DNA was detected in the same sample by PCR with a mean Cq of 33, although it was not identified by HTS.

In the case of the *G. portentosa* meal (sample 76), *T. molitor* DNA was again detected by PCR, along with traces of *A. diaperinus* DNA. Interestingly, HTS analysis also revealed the presence of *G. portentosa* in the *H. illucens* meal (sample 64) from the same producer, suggesting possible cross-contamination during rearing or processing.

As observed in the previous results, some discrepancies between the two analytical methods, PCR and HTS, can be noted regarding contaminant species, which may be attributed to the higher sensitivity of PCR.

However, the untargeted HTS approach also detected two beetle species, *T. castaneum* and *N. rufipes*, each with relative abundances exceeding 10%, in sample 70. Rajendran and Parveen (2005) [76] reported these species as insect pests attacking various animal products. In sample 71, two globally widespread fly species were detected: *C. megacephala*, and *Musca domestica*. In sample 67, *Sitophilus zeamais* (Motschulsky), a beetle known as a severe internal primary pest of stored products due to its high reproductive capacity and short life cycle [77], was detected. It should be noted that the latter species exhibited an extremely low relative abundance.

The 21 single-species insect meal samples were also analyzed using official PCR methods developed for the detection of animal proteins in feed (Table 11). Of these 21 samples, 19 originated from insect species authorized in the EU for inclusion in fish, pig, and poultry feed. These could therefore potentially be used as raw materials in such feeds and be subjected to official monitoring and control. Accordingly, they were analyzed using the relevant official PCR protocols (Table 11).

The results showed that no ruminant, porcine, or poultry DNA was detected in 13 of the 19 samples. Among the six remaining samples, five tested positive for ruminant DNA. One of these also contained poultry DNA, and another tested positive for both porcine and poultry DNA. The sixth sample contained only poultry DNA. Of these six samples, five originated from Europe (including four from the Netherlands), and one originated from Canada.

Among the three samples that tested positive exclusively for ruminant DNA (samples 61, 70 and 72), the mean Cq values ranged from 33 to 36 cycles, which are among the latest signals observed. A mean Cq value of 36 cycles for the ruminant target is very close to the threshold (cut-off value at 36.6 cycles) at which a sample is considered positive. According to the EURL-AP SOPs [60,61,62], specific cut-off Cq values have been established to determine the positive or negative result of an amplification curve.

For the other two samples, samples 62 and 67, that tested positive for ruminant DNA with earlier Cq values (below 33 cycles), both also showed the presence of poultry DNA, and sample 67 also tested positive for porcine DNA. Sample 62 showed an early poultry DNA signal (mean Cq of 25 cycles), as did sample 63 (mean Cq of 19 cycles), which originated from the same producer.

Since insect meals are generally included in animal feed at low incorporation rates (typically a few percent), any potential contamination from the insects’ own feed would be diluted in the final feed. Thus, most of the PCR signals detected in the undiluted insect meals would no longer be detectable in multi-ingredient feed. However, samples 63 and 62 showed very early Cq values for poultry DNA (mean Cq of 19 and 25 cycles). At such low Cq values, the presence of unauthorized poultry-derived products such as meat waste is strongly suspected. This is particularly relevant since DNA extraction from eggs (authorized product) gives a low yield of collected DNA [78]. Moreover, even at low incorporation rates, 1% for example, poultry DNA would still be detectable by PCR, which could pose a compliance issue under the current official analytical framework if these feeds also contain porcine PAPs and are intended for poultry.

### 3.4. Feed: Multi-Ingredient Samples

Given the restrictions on the substrates allowed for insect rearing, the production cost of insect meals remains relatively high, preventing them from gaining significant market share as raw materials in livestock feed formulations. As a result, we were unable to obtain any multi-ingredient feed for livestock containing insect-derived ingredients. Instead, insect meals are predominantly used in pet food products. In this category, we collected 18 dog foods (from 11 different brands), 5 cat foods (from 4 brands), 3 products intended for both cats and dogs (from the same brand), 13 feeds for ornamental fish or fishing bait (from 8 brands), 2 reptile feeds (from 2 brands), 1 turtle feed, and 1 feed for backyard hens and poultry. This represents a total of 43 multi-ingredient feeds (samples 77 to 119) from 22 different brands labeled as containing insect ingredients. Among these, 8 products listed multiple insect species. In total, we recorded 20 samples containing *H. illucens*, 16 with *T. molitor*, 10 with *B. mori*, 1 with *A. domesticus*, 1 with *Gryllus testaceus* (Walker), 3 labeled generically as “crickets”, and 4 labeled only as “insects” without species-level identification.

In the analysis of these multi-ingredient pet food samples, PCR inhibition was observed more frequently. To overcome this and enable the detection of the species present, additional dilutions (up to 160-fold) were tested. These dilutions were assessed for all PCR targets. For these samples, the reported Cq values correspond to those obtained under conditions where inhibition was completely removed for the PCR test concerned (a letter referring to the dilution used is indicated in the Table 12, Table 14 and Table 15).

#### 3.4.1. Results on Multi-Ingredient Feed Samples Labeled as Containing a Single Insect Species

Results on multi-ingredient feed samples labeled as containing a single insect species are presented in Table 12. Among the 14 samples labeled as containing *H. illucens*, DNA from this species was effectively detected in 13 of them. In two of these cases, DNA from a second insect species was also identified. For sample 88, *A. domesticus* DNA was detected with a late Cq value (mean Cq of 38 cycles), suggesting potential contamination. In contrast, sample 89 showed the presence of *T. molitor* DNA with a mean Cq of 30 cycles, a value incompatible with simple contamination. In the remaining sample, sample 90, *T. molitor* DNA was detected instead of *H. illucens*.

Examination of ingredient lists reveals that certain samples—especially those from the same brand—share nearly identical compositions (e.g., samples 87 and 88). Interestingly, some products from different brands also have identical compositions. For instance, samples 89 and 90 list the same ingredients, with potential variation only in the proportions that are not disclosed. Both of these samples contain *T. molitor*, which was confirmed by HTS, despite not being labeled as such. *H. illucens* was also detected in sample 89, and it presented the latest Cq value among all 13 *H. illucens*-positive samples, indicating a low quantity.

Regarding the 10 samples labeled as containing *T. molitor*, *T. molitor* DNA was detected in 9. Among those, 6 samples also tested positive—though at lower levels—for *H. illucens* and/or *A. diaperinus*. In the 10th sample (sample 100), which claimed to contain 40% *T. molitor*, only *H. illucens* DNA was detected. Due to PCR inhibition observed in this sample, serial dilutions (1, 2, 5, 10, 20, 40, 80 and 160 fold) were performed to mitigate the effect. Inhibition was completely lifted at the 40 fold dilution. All dilutions were tested with all PCR targets, and only *H. illucens* was detected.

For the 5 samples labeled as containing *B. mori*, three also showed the presence of either *T. molitor* or *H. illucens*, in addition to *B. mori*. The remaining two samples (samples 104 and 105) did not contain *B. mori* either; instead, both revealed the presence of *T. molitor* and *H. illucens*. These two samples had previously been analyzed using both light microscopy (LM) and high-throughput sequencing (HTS) in the study by Marien et al. [45], which confirmed the absence of *B. mori* and the presence of *T. molitor* in both samples, as well as the presence of *H. illucens* in sample 104. For sample 105, *H. illucens* DNA produced a late Cq signal in the PCR assay, suggesting a low DNA quantity. Since the PCR assay targets a multicopy DNA fragment, this low level may not have been detectable by LM. In HTS, the signal for *H. illucens* may have been masked by the higher abundance of *T. molitor* DNA.

Finally, two samples labeled as containing ‘cricket’ (samples 106 and 107) without specifying the species did not yield any detection for the two tested cricket species, including *A. domesticus*, which, as previously noted, is the most commonly used species when ‘cricket’ is mentioned. However, *T. molitor* DNA was found in both samples. These results were further confirmed by HTS. Notably, these products, although from different brands, share identical ingredient lists.

Once again, HTS results revealed the presence of insect species classified as storage pests in certain samples. *Rhyzopertha dominica* (F.) was detected in samples 85 and 86, both from the same brand (brand 42) and sharing a highly similar composition. This species is considered one of the most important pests of stored wheat and other crops such as barley, beans, chickpeas, millet, oats, sorghum, rice, etc. [79], as well as various animal-derived products [76]. *Sitophilus oryzae* (L.) was identified in sample 92, in which sorghum appears as the second ingredient. This species is well known to infest stored sorghum grains [80]. Similarly, *Cryptolestes ferrugineus* (Stephens), also detected, is regarded as a major threat to the global food supply chain due to its economic impact and contribution to food waste [81]. Finally, in sample 103, *Daphnia pulex* (Leydig) was detected at a very low read abundance (0.6%). This aquatic species aligns with the presence of several marine-derived ingredients in the sample, including salmon meal, krill meal, and *Gammarus* (F.).

**Table 12:** Results obtained by real-time PCR tests and high-throughput sequencing (HTS, i.e., metabarcoding) on 31 multi-ingredient feed samples for which the insect species was specified and only a single species was present. PCR results are based on undiluted extracts, except when marked with a letter referring to a dilution used. For positive results, mean Cq values obtained from 2 DNA extracts are given in brackets. Expected PCR positive results are marked with a gray background. Potentially expected results in cases where the species is not indicated are marked with a blue background. (Real-time PCR settings on LightCycler 480 were set to second derivative max and high confidence). HTS results represent the relative read abundance (%) of each species detected based on *COI*, *12S*, and *28S* metabarcoding. Percentages indicate the proportion of total DNA barcode reads assigned to each species per sample, averaged across the results provided by the three metabarcoding markers.

		Results with PCR Tests for the Detection of						
Sample No.	Expected Species	*T. molitor*	*H. illucens*	*A. diaperinus*	*B. mori*	*A. domesticus*	*G. sigillatus*	HTS
77	*H. illucens*	-	+(22) ^a^	-	-	-	-	*H. illucens* (100%)
78	*H. illucens*	-	+(19)	-	-	-	-	*H. illucens* (100%)
79	*H. illucens*	-	+(21)	-	-	-	-	*H. illucens* (100%)
80	*H. illucens*	-	+(19) ^a^	-	-	-	-	*H. illucens* (100%)
81	*H. illucens*	-	+(26) ^a^	-	-	-	-	*H. illucens* (100%)
82	*H. illucens*	-	+(23) ^a^	-	-	-	-	*H. illucens* (100%)
83	*H. illucens*	-	+(20) ^a^	-	-	-	-	*H. illucens* (100%)
84	*H. illucens*	-	+(20)	-	-	-	-	*H. illucens* (100%)
85	*H. illucens*	-	+(22)	-	-	-	-	*H. illucens* (99.8%), *Rhyzopertha dominica* (0.2%)
86	*H. illucens*	-	+(25) ^a^	-	-	-	-	*H. illucens* (98.3%), *Rhyzopertha dominica* (1.7%)
87	*H. illucens*	-	+(21)	-	-	-	-	*H. illucens* (100%)
88	*H. illucens*	-	+(23) ^a^	-	-	+(38)	-	*H. illucens* (100%)
89	*H. illucens*	+(30)	+(34)	-	-	-	-	*T. molitor* (100%)
90	*H. illucens*	+(32)	-	-	-	-	-	*T. molitor* (100%)
91	*T. molitor*	+(28)	-	-	-	-	-	*T. molitor* (100%)
92	*T. molitor*	+(28)	-	-	-	-	-	*T. molitor* (91.0%), *Sitophilus oryzae* (6.4%), *Cryptolestes ferrugineus* (2.6%)
93	*T. molitor*	+(25)	-	-	-	-	-	*T. molitor* (100%)
94	*T. molitor*	+(31)	-	-	-	-	-	*T. molitor* (100%)
95	*T. molitor*	+(30)	+(32)	-	-	-	-	*T. molitor* (100%)
96	*T. molitor*	+(27)	+(33)	+(36)	-	-	-	*T. molitor* (100%)
97	*T. molitor*	+(26)	-	+(38)	-	-	-	*T. molitor* (100%)
98	*T. molitor*	+(27)	-	+(38)	-	-	-	*T. molitor* (100%)
99	*T. molitor*	+(27)	-	+(40)	-	-	-	*T. molitor* (100%)
100	*T. molitor*	-	+(26) ^c^	-	-	-	-	*H. illucens* (100%)
101	*B. mori*	+(38)	-	-	+(26) ^a^	-	-	*B. mori* (99.6%), *T. molitor* (0.4%),
102	*B. mori*	-	+(30)	-	+(29)	-	-	*B. mori* (99.6%)
103	*B. mori*	-	+(38) ^b^	-	+(30) ^b^	-	-	*B. mori* (99.4%), *Daphnia pulex* (0.6%)
104	*B. mori*	+(33)	+(23)	-	-	-	-	*T. molitor* (77.8%), *H. illucens* (21.9%), *Sitophilus oryzae* (0.2%)
105	*B. mori*	+(30)	+(36)	-	-	-	-	*T. molitor* (100%)
106	Cricket	+(31)	-	-	-	-	-	*T. molitor* (100%)
107	Cricket	+(34)	-	-	-	-	-	*T. molitor* (100%)

^a^ = 10 fold dilution of extracts DNA, ^b^ = 20 fold dilution of extracts DNA, ^c^ = 40 fold dilution of extracts DNA.

These results also highlight that products from the same brand, although labeled as containing different insect species, exhibit highly similar DNA profiles. This is particularly evident for the four products of brand 44 (samples 89, 95, 105 and 106), each labeled as containing a different single insect species; *H. illucens*, *T. molitor*, *B. mori*, and cricket, respectively. Despite these distinctions, all four samples yielded comparable results for *T. molitor* DNA, and three of them also tested positive for *H. illucens* (Table 13).

This was also the case for four products from brand 45 (samples 90, 94, 104 and 107), also labeled, respectively, as containing *H. illucens*, *T. molitor*, *B. mori*, and cricket. Here again, *T. molitor* DNA was consistently detected across all samples, while *H. illucens* was detected in only one sample, the one labeled as containing *B. mori* (Table 13).

The overall HTS results on these samples support the presence of *T. molitor* in all of them. Regarding the detection of *H. illucens*, as previously explained, the sensitivity of this test appears to be higher than that of the HTS assay, which explains why only sample 104—showing a Cq value of 23 cycles with the *H. illucens*-specific PCR target—was detected by HTS.

Furthermore, it can also be noted that these two brands offer an identical range of products based on the list of raw materials.

#### 3.4.2. Results for Multi-Ingredient Feed Samples Labeled as Containing Insects Without Species Specification

For certain multi-ingredient feeds, the label only indicates the presence of insects without specifying the species (Table 14). Four such samples were analyzed, for which no information regarding the insect species could be found either on the packaging or through the manufacturers’ websites. In all four cases, *H. illucens* was the only species detected by PCR and HTS.

#### 3.4.3. Results for Multi-Ingredient Feed Samples Labeled as Containing Multiple Insect Species

Regarding the eight samples labeled as containing multiple insect species, Table 15 reveals several discrepancies between the declared species composition and the analytical results. Only three of the samples matched the expected results. However, in one of these three cases—sample 114—the DNA content of *T. molitor* was very low (Cq value of 39 cycles), despite this species being listed as the main insect ingredient (13% mealworm meal, 7% silkworm pupae meal, and 5% *H. illucens* larvae meal).

For two other samples (115 and 116), an additional insect species was detected by PCR that was not listed on the label. In both cases, *H. illucens* was identified alongside the declared species.

In sample 115, the product is labeled as containing *G. testaceus*, but this species was not detected by HTS. Instead, three other species from the *Gryllidae* family were identified, including *Teleogryllus emma* (Ohmachi & Matsuura), which accounted for 20.2% of the reads, and is reared in Asia [74]. This species was also detected in sample 116, with a read assignment rate of 6.2%.

In the remaining three samples, at least one of the labeled insect species was not detected. Specifically, the declared compositions of samples 117, 118 and 119 were found to be inaccurate and potentially misleading for consumers. Sample 117 is labeled to contain 15% each of *B. mori*, *H. illucens*, and *T. molitor*, but *T. molitor* was not detected by any of the methods used (real-time PCR, high-throughput sequencing, or light microscopy). These results were previously reported in Marien et al. [45]. Sample 118 is labeled as containing 15% *T. molitor* and 10% *H. illucens*, but only *H. illucens* was detected by PCR and HTS. For sample 119, the composition lists *H. illucens*, *B. mori*, *T. molitor*, and crickets (in that order), but only *H. illucens*, the most abundant declared insect, was detected by both methods.

## 4. Discussion

As demonstrated by the overall results, whether using PCR or HTS, the most abundant species detected was consistently the same across both analytical methods. However, discrepancies were noted in the detection of contaminants or low-abundance species. These differences may be partly explained by the DNA extraction protocols used, which varied in accordance with methodological requirements. Since the samples were also analyzed using the official PCR methods for detecting animal proteins in livestock feed, DNA extractions were performed using the method recommended by the EURL-AP. These extracts were used for all real-time PCR analyses. In contrast, the DNA used for HTS was obtained using the CTAB method, which allows for accurate DNA quantification and may yield different extraction efficiencies compared to the EURL-AP protocol. In addition to extraction methods, intrinsic differences between the two analytical approaches likely contributed to the observed discrepancies. PCR, as a targeted method, offers high sensitivity for specific targets (especially when assays target multi-copy genes), which is crucial for identifying low-abundance contaminants or verifying species authenticity. However, given the rapid expansion and diversification of farmed insect species, real-time PCR methods face inherent limitations, as they rely on predefined, species-specific primers and probes. On the other hand, the untargeted nature of HTS through metabarcoding enables the detection of a broader range of species, including unexpected or unauthorized taxa, without prior knowledge of their presence. However, HTS approaches depend heavily on comprehensive and well-curated reference databases for accurate taxonomic assignments, and gaps in these databases can lead to ambiguous or incomplete identifications. Moreover, HTS sensitivity is generally lower compared to real-time PCR. Therefore, combining both targeted (real-time PCR) and untargeted (HTS) methods represents the most robust approach for regulatory bodies and quality control laboratories. PCR can offer rapid and highly sensitive detection of known, prioritized species, while HTS can continuously screen products broadly, ensuring adaptability to the evolving species landscape and promoting compliance within the insect-based product industry.

The results obtained across all samples indicate a high occurrence of cross-contamination when producers manufacture products based on different insect species. Most cases of cross-contamination are not problematic, as the contaminating species are also authorized for use in the relevant food or feed applications. However, a few instances are more concerning, particularly when the contaminating species is not authorized under current legislation. Insect-based ingredients or raw materials are often not produced by the companies that incorporate them into finished products. Moreover, insect producers are not always the same entities as those responsible for processing the insects into flour. It also appears that, unlike in previous years when the vast majority of insect farmers managed all life stages of the insect species they produced (so-called “full-liners”), recent developments in the sector have led to a decentralization of the production chain. A growing number of companies now specialize in specific segments of the production process, such as reproduction, delivery of neonates, rearing, or processing. While decentralized insect operation models offer advantages by reducing investment risks and operational complexity [11], they may also increase the number of potential points for cross-contamination across the supply chain, including during transport. As we did not have access to rearing or processing facilities, it is not possible to determine whether cross-contamination occurred during insect breeding, handling, or further down the processing line. Such contamination may arise if production lines are not dedicated to a single insect species or are insufficiently cleaned between productions of different insects.

The results also revealed discrepancies between the labeled and detected species in certain products. Most of these mismatches were observed in multi-ingredient pet food products—though this is also the category from which the largest number of samples was obtained. This highlights the importance of species authentication testing, particularly if the insect production sector continues to expand.

Finally, the results obtained using the official PCR methods for detecting animal proteins in livestock feed show that, in the majority of pure single-species insect meals (whether intended for feed or food), no ruminant, porcine, or poultry DNA was detected. It was relevant to assess the current market products, as in some cases, the use of substrates containing animal-derived DNA, even when authorized, could pose regulatory problems. For instance, if an insect meal is produced from insects reared on a milk-containing substrate, remnants of that substrate in the insects’ digestive tract could end up in the final meal. Since milk is a source of ruminant DNA, this meal could test positive in the official ruminant PCR assay. If such a meal was then used as a raw material in poultry feed that also contains authorized porcine processed animal proteins (PAPs), the presence of ruminant DNA could lead to regulatory non-compliance, as ruminant PAPs are strictly prohibited in livestock feed due to the associated risk of BSE. To fill in the analytical gaps still existing and identify the origin of PCR signals, a mass spectrometry method is being developed to determine the proteins present in the sample [82,83]. The information provided by this complementary method should discard cases of unauthorized animal ingredients in the substrates.

In some single-species insect meals, DNA from ruminants, pigs, and/or poultry was detected. Nevertheless, these meals would likely not pose any problem as long as the insects were reared on substrates containing only authorized animal ingredients (e.g., milk and egg products). Indeed, according to the present EURL-AP SOP on Operational protocol for the combination of light microscopy and PCR, these samples would be analyzed firstly by light microscopy which would not detect any particles from forbidden materials. Consequently, no additional PCR analysis would be performed. Moreover, in a multi-ingredient feed, the presence of residual DNA—potentially derived from an authorized substrate would be diluted and probably below the limit of detection.

However, in two pure insect meal samples (samples 63 and 62), poultry DNA was detected at levels such that, even at an inclusion rate of just 1% in feed, the presence of poultry DNA would still be detectable. This would be problematic if these meals were used in poultry feed that also contains porcine PAPs. Furthermore, in the case of these two meals, the strong poultry DNA signal raises suspicion about the possible use of unauthorized poultry-derived products, such as meat waste, in the rearing substrate, since egg products, which are permitted in insect feed, generally contain only low quantities of DNA.

## 5. Conclusions

This study highlights key challenges in ensuring authenticity and regulatory compliance in the expanding insect-based food and feed industry. Due to industrial processing, visual identification of insect species in final products is unfeasible, necessitating dedicated authentication methods. In this context, both real-time PCR and high-throughput sequencing (metabarcoding) proved to be highly complementary approaches for species identification. Real-time PCR enables rapid and sensitive detection of specific, targeted species, while HTS offers a broader overview of the taxonomic composition, including the ability to detect non-target or unexpected species.

Among the 119 commercial samples analyzed, both methods generally produced concordant results, allowing accurate authentication of the labeled insect species. Their respective strengths provided additional insights: real-time PCR was particularly effective for detecting low-level contamination by targeted species, whereas HTS identified the presence of non-target insect DNA, including potential storage pests not covered by the PCR screening panel. This complementarity strengthens the overall reliability of species identification and represents a valuable tool for quality control of insect-based products.

Cross-contaminations were more frequently observed when producers handled multiple insect species simultaneously. In 10 out of the 119 samples analyzed, the expected species was not detected, while other insect species were present, cases likely resulting from labeling errors or intentional misrepresentation. Furthermore, pests of stored products or unauthorized species were identified in 13 of the 119 samples.

Analyses of pure insect flours and meals using official PCR tests outlined in the SOPs of the EURL-AP showed that, although some use of animal-derived substrates is legally permitted, a subset of samples (8 of 28 food samples and 6 of 21 feed samples) tested positive for ruminant, porcine, or poultry DNA. Notably, strong poultry DNA signals in two feed samples raise serious concerns regarding the potential use of unauthorized animal by-products as rearing substrates.

These findings underscore the urgent need for robust DNA-based authentication methods to support official controls of the European regulatory framework, particularly regarding insect species composition, rearing practices, and accurate labeling, ultimately contributing to the safety and transparency of insect-based products.

## Figures and Tables

**Table 1:** Information on samples of insect pure flour intended for food.

Sample No.	Expected Species	Kind of Product	Brand No.	Origin	Composition	Claims
1	*T. molitor*	Powder of molitor	1	France	Dehydrated whole mealworms powder (*Tenebrio molitor*)	“*Tenebrio molitor*” in the composition
2	*T. molitor*	Insect powder	2	Portugal	100% *Tenebrio molitor* larvae (yellow mealworms)	“*Tenebrio molitor*” in the composition
3	*T. molitor*	Roasted and ground mealworms	3	Austria	100% heat-treated mealworms	“*Tenebrio molitor* from Austrian breeding” in the composition
4	*T. molitor*	Dehydrated insect powder (*Tenebrio molitor*)	4	France	100% *Tenebrio molitor*	“*Tenebrio molitor*” in the composition and in the name of product
5	*T. molitor*	Mealworm meal—heat dried	5	Denmark	Mealworm	“Mealworm” in the name of product
6	*T. molitor*	Mealworm meal—freeze-dried	5	Denmark	Mealworm	“Mealworm” in the name of product
7	*T. molitor*	Mealworm meal—freeze-dried	5	Denmark	*Tenebrio molitor*	“*Tenebrio molitor*” in the composition
8	*T. molitor*	Mealworm—insect meal	6	Germany	100% dried and ground mealworms (lat. *Tenebrio molitor*)	“*Tenebrio molitor*” in the composition
9	*T. molitor*	Dehydrated *Tenebrio molitor* (flour)	7	Belgium	Dehydrated *Tenebrio molitor*	“*Tenebrio molitor*” in the composition
10	*T. molitor*	Mealworms powder	8	Belgium	Mealworm meal	“Mealworms” in the name of product
11	*A. domesticus*	Dried grounded insects, *Acheta domesticus*	9	Lithuania	100% dried grounded insects, domestic crickets *Acheta domesticus*	“*Acheta domesticus*” in the composition
12	*A. domesticus*	100% cricket powder	10	Belgium	100% cricket powder (*Acheta domesticus*)	“*Acheta domesticus*” in the composition
13	*A. domesticus*	Cricket flour	11	New Zealand	Crickets (*Acheta domesticus*) 100%	“*Acheta domesticus*” in the composition
14	*A. domesticus*	Crickets—insect meal	6	Germany	100% dried and ground crickets (lat. *Acheta domesticus*)	“*Acheta domesticus*” in the composition
15	*A. domesticus*	House cricket whole freeze-dried	5	Denmark	House cricket whole freeze-dried	“*Acheta domesticus*” in the composition
16	*A. domesticus*	Insect meal, ground crickets	12	Germany	Insect meal (ready to eat) made from dried, ground crickets (house crickets—*Acheta domesticus*)	“*Acheta domesticus*” in the composition
17	*A. domesticus*	Cricket protein powder	13	United Kingdom	House cricket (*Acheta domesticus*) 100%	“*Acheta domesticus*” in the composition
18	*A. diaperinus*	Buffalo worm meal	14	Austria	100% buffalo worm meal (*Alphitobius diaperinus*) freeze-dried and ground	“*Alphitobius diaperinus*” in the composition
19	*A. diaperinus*	Buffalo larvae freeze-dried (*Alphitobius*)	5	Denmark	Buffalo larvae freeze-dried (*Alphitobius*)	“*Alphitobius*” in the name of product
20	*A. diaperinus*	Flour	15	The Netherlands	Lesser mealworm—*Alphitobius diaperinus*	“*Alphitobius diaperinus*” in the composition
21	*A. diaperinus*	Flour	15	The Netherlands	*Alphitobius diaperinus* Powder—Lesser mealworm	“*Alphitobius diaperinus*” in the composition
22	*G. sigillatus*	Flour	16	The Netherlands	Banded Crickets Powder—*Gryllodes sigillatus*	“*Gryllodes sigillatus*” in the composition
23	*G. sigillatus*	Freeze-dried cricket (*Gryllodes sigillatus*)	5	Denmark	*Gryllodes sigillatus*	“*Gryllodes sigillatus*” in the name of product
24	*L. migratoria*	Locust flour freeze-dried (*Locusta migratoria*)	5	Denmark	*Locusta migratoria*	“*Locusta migratoria*” in the name of product
25	Cricket	Cricket powder	8	Belgium	Cricket powder	“Cricket” in the name of product
26	Cricket	Cricket powder	17	USA	Cricket powder	“Cricket” in the composition
27	Cricket	Cricket powder	17	USA	Cricket powder	“Cricket” in the composition
28	Cricket	Prebiotic protein powder	18	USA	100% cricket	“Cricket” mentionned on the label

**Table 2:** Information on samples of multi-ingredient food containing insects.

Sample No.	Expected Species	Kind of Product	Brand No.	Origin	Composition	Claims
29	*T. molitor*	Granola	2	Portugal	Oats, chocolate chips, honey, dried *Tenebrio molitor* larvae (yellow mealworms) (gluten), sunflower seeds, cocoa powder, coconut oil, salt	“*Tenebrio molitor*” in the composition
30	*T. molitor*	Aperitif product	2	Portugal	Wheat flour (gluten), water, pea protein isolate, olive oil, dried *Tenebrio molitor* larva (yellow mealworm) (gluten), rosemary 1%, salt, paprika, black pepper	“*Tenebrio molitor*” in the composition
31	*T. molitor*	Cereal bar	2	Portugal	Dates, almonds, oats, *Tenebrio molitor* larvae (yellow mealworms) 7.5% (gluten), hydrolyzed collagen, dried apple 1%, cinnamon 1%, sea salt, natural flavoring	“*Tenebrio molitor*” in the composition
32	*T. molitor*	Pasta	2	Portugal	Durum wheat semolina (gluten), *Tenebrio molitor* larvae (yellow mealworms) 10% (gluten), soy protein	“*Tenebrio molitor*” in the composition
33	*T. molitor*	Bread baking mix	3	Austria	Wheat protein, wholemeal spelled flour, mealworm powder 10%, tartar baking powder (cream of tartar, sodium bicarbonate, starch), linseed, dried wheat sourdough, psyllium husk, salt, barley malt, spices	“Mealworms” in the composition
34	*T. molitor*	Pasta	19	Belgium	Fine durum wheat semolina (gluten), eggs, *Tenebrio molitor* flour 10% (dehydrated and ground mealworms)	“*Tenebrio molitor*” in the composition
35	*T. molitor*	Cereal bar	3	Austria	Honey 30%, oat flakes, almonds, butter, puffed spelled, dried mealworms 5%, linseed, cinnamon	“Mealworms” in the composition
36	*A. domesticus*	Cereal bar	20	Germany	Oat flakes 18%, rice syrup, cornflakes (maize) 16%, cranberries (cranberries, apple concentrate, sunflower oil, rice flour), roasted hazelnuts, *Acheta domesticus* (house cricket) powder 10.5%, coconut oil, apple pieces	“*Acheta domesticus”* in the composition
37	*A. domesticus*	Aperitif product	20	Germany	Pea flour 61%, lentil flour 10%, garlic seasoning preparation 8.4% [dried vegetables 81% (garlic 75%, onion), yeast extract, spices, spice extracts], *Acheta domesticus* (house cricket) powder 7%, poppy seeds, pumpkin seed flour, potato starch, salt, rapeseed oil	“*Acheta domesticus”* in the composition
38	*A. domesticus*	Minced meat substitute	20	Germany	Pea protein 70%, *Acheta domesticus* (house cricket) powder 20%, broad bean 10%	“*Acheta domesticus”* in the composition
39	*A. domesticus*	Burger mix	12	Germany	Protein product 40% (pea protein 28%, cricket powder (house cricket (*Acheta domesticus*)) 8%, broad bean protein 4%), flour (broad bean, linseed), rapeseed oil, spices, broth (sea salt, maltodextrin, yeast extract, non-hydrogenated sunflower oil, caramelized sugar syrup, lovage, mace, pepper), onions, salt, shiitake mushroom powder, smoked salt, beetroot powder	“*Acheta domesticus”* in the composition
40	*A. domesticus*	Minced meat substitute	12	Germany	Pea protein 70%, cricket powder (house cricket (*Acheta domesticus*)) 20%, ground broad beans 10%	“*Acheta domesticus”* in the composition
41	*A. domesticus*	Granola	10	Belgium	Oat flakes, maple syrop, hazelnuts 6%, agave syrup, sunflower oil, cricket powder *Acheta domesticus* 5%, pumpkin seeds, quinoa, sunflower seeds, flax seeds, sesame seeds, cinnamon, nutmeg, salt	“*Acheta domesticus”* in the composition
42	*A. domesticus*	Cereal bar	10	Belgium	Hazelnuts 26%, agave syrup, pumpkin seeds 12%, oat flakes, quinoa, rice syrup, sesame seeds, buckwheat, cricket powder *Acheta domesticus* 5%, hemp seeds, flax seeds	“*Acheta domesticus”* in the composition
43	*A. domesticus*	Cereal	13	Germany	Sprouted oat flakes 56%, criket flour (*Acheta domesticus*) 10%, dried sprouted red quinoa flakes 9%, panela, rice powder (rice syrup powder, rice starch, rice flour), dried apples 6%, apple powder 5%, sea salt, natural flavoring, ground cinnamon 0.1%	“*Acheta domesticus”* in the composition
44	*A. domesticus*	Aperitif product	13	Germany	Pea flour 75%, cricket flour (*Acheta domesticus*) 10%, garlic 7%, sunflower oil, herbs 3.2% (wild garlic, paprika, satureja, basil, pepper, oregano, thyme), potato starch, yeast extract, salt	“*Acheta domesticus”* in the composition
45	*A. domesticus*	Pasta	13	United Kingdom	Red lentil flour 90%, cricket flour (*Acheta domesticus*) 10%	“*Acheta domesticus”* in the composition
46	*A. domesticus*	Aperitif product	2	Portugal	*Acheta domesticus* (house cricket) dried 68.5%, olive oil, thyme 3.9%, oregano, marine salt, garlic (sulfites), paprika, onion, cayenne pepper 0.2%	“*Acheta domesticus”* in the composition
47	*A. diaperinus*	Energy bar	21	Germany	Isomaltulose, sesame 38%, diced almonds 12%, freeze-dried buffalo worms (*Alphitobius diaperinus*) 10%	“*Alphitobius diaperinus”* in the composition
48	*A. diaperinus*	Aperitif product	6	Germany	100% dried buffalo worms (Latin: *Alphitobius diaperinus*)	“*Alphitobius diaperinus”* in the composition
49	*A. diaperinus*	Granola	1	France	Oat flakes 55.6%, rice syrup, pumpkin seeds, dried apple 5.7%, puffed rice, whole Buffalo worms (*Alphitobius diaperinus*) powder 5%, honey, rapeseed oil, coconut blossom sugar, pea protein, vanilla extract 1.6%	“*Alphitobius diaperinus”* in the composition
50	*A. diaperinus*	Pasta	1	France	Durum wheat semolina, powdered whole Buffalo worm (*Alphitobius diaperinus*) 3.85%, dehydrated egg white.	“*Alphitobius diaperinus”* in the composition
51	*A. diaperinus*	Protein bar	1	France	Apricot paste 25.5%, almonds 24%, date paste, unshelled almond puree, pea protein, sunflower protein, whole buffalo worms (*Alphitobius diaperinus*) powder 5.5%, honey, chia seeds, concentrated apricot juice, natural flavoring	“*Alphitobius diaperinus”* in the composition
52	*A. diaperinus*	Cereal bar	22	Germany	Oat flakes 21%, protein blend (insect protein (buffalo worm, 12%), pea protein isolate), isomaltooligosaccharide (prebiotic fiber from tapioca starch), honey, macadamia nuts 6%, almonds, cranberries 7%, chocolate coating (cocoa mass, sugar, cocoa butter), salt, natural flavorings, sweetener (sucralose)	“Buffalo worm” in the composition
53	Cricket	Pasta	19	Belgium	Fine durum wheat semolina (gluten), hemp flour 9%, and cricket flour 3% (dehydrated and ground)	“Cricket” in the composition
54	Cricket	Aperitif product	8	Belgium	Wheat flour, water, sunflower oil, potato flakes, dried glucose syrop, cricket powder, salt	“Cricket” in the composition
55	Cricket	Aperitif product	8	Belgium	Pea powder, cricket powder 10%, poppy powder, lentil flour, pumpkin seed powder, rapeseed oil, spices, yeast, starch, sea salt	“Cricket” in the composition

**Table 3:** Information on samples of pure meal intended for feed.

Sample No.	Expected Species	Kind of Product	Brand No.	Origin	Composition	Claims
56	*H. illucens*	Meal for feed	23	Tunisia	*Hermetia illucens* meal	“*Hermetia illucens*” mentionned on the label
57	*H. illucens*	Meal for feed	24	France	*Hermetia illucens*	“*Hermetia illucens*” mentionned on the label
58	*H. illucens*	Meal for feed	25	France	*Hermetia illucens*	“*Hermetia illucens*” mentionned on the label
59	*H. illucens*	Meal for feed	26	France	*Hermetia illucens*	“*Hermetia illucens*” mentionned on the label
60	*H. illucens*	Meal for feed	26	France	*Hermetia illucens*	“*Hermetia illucens*” mentionned on the label
61	*H. illucens*	Meal for pet food	27	Germany	Insects (*Hermetia illucens*) 100%	“*Hermetia illucens*” in the composition
62	*H. illucens*	Meal for feed	28	The Netherlands	*Hermetia illucens*	“*Hermetia illucens*” mentionned on the label
63	*H. illucens*	Meal for feed	28	The Netherlands	*Hermetia illucens*	“*Hermetia illucens*” mentionned on the label
64	*H. illucens*	Meal for feed	5	Denmark	Black soldier fly meal	“*Black soldier fly*” in the name of product
65	*T. molitor*	Meal for feed	29	France	*Tenebrio molitor*	“*Tenebrio molitor*” mentionned on the label
66	*T. molitor*	Meal for feed	29	France	*Tenebrio molitor*	“*Tenebrio molitor*” mentionned on the label
67	*T. molitor*	Meal for pet food	30	The Netherlands	Mealworm flour	“Mealworm” in the name of product
68	*T. molitor*	Meal for feed	31	Poland	*Tenebrio molitor*	“*Tenebrio molitor*” mentionned on the label
69	*T. molitor*	Meal for ornamental birds	32	Spain	Protein flour from *Tenebrio molitor* larvae	“*Tenebrio molitor*” in the composition
70	*B. mori*	Meal for ornemental fish	33	The Netherlands	Silkworm flour	“Silkworm” mentionned on the label
71	*B. mori*	Meal for fishing bait	34	The Netherlands	Silkworm protein meal	“Silkworm” mentionned on the label
72	Cricket	Meal for pet food	35	Canada	Roasted whole Canadian cricket powder	“Cricket” mentionned on the website
73	*G. assimilis*	Meal for feed	36	Spain	*Gryllus assimilis*	“*Gryllus assimilis*” mentionned on the label
74	*G. assimilis*	Meal for feed	5	Denmark	*Gryllus assimilis*	“*Gryllus assimilis*” in the information mentioned on the site
75	*Blabtica dubia*	Meal for pet food	5	Denmark	Freeze-dried Dubia cockroach meal	“*Blabtica dubia*” in the information mentioned on the site
76	*Gromphadorhina portentosa*	Meal for pet food	5	Denmark	Madagascar Cockroach Meal	“*Gromphadorhina portentosa*” in the information mentioned on the site

**Table 4:** Information on samples of multi-ingredient feed.

Sample No.	Expected Species	Kind of Product	Brand No.	Origin	Composition	Claims
77	*H. illucens*	Pet food for dogs	27	Germany	Rice 37%, insects (*Hermetia illucens*) 20%, pregelatinized rice flour 20%, rice protein 11%, hemp oil 2.5%, linseed 2%, carrot 1%, potassium chloride 0.75%, sodium chloride 0.35%, algae powder 0.27%	“*Hermetia illucens*” in the composition
78	*H. illucens*	Pet food for dogs	27	Germany	Insects (*Hermetia illucens*) 25%, sweet potato, pea starch, pea protein, linseed, rapeseed oil, brewer’s yeast, sunflower oil, algal lime	“*Hermetia illucens*” in the composition
79	*H. illucens*	Pet food for dogs	37	Germany	Insects, dried (black soldier fly), sweet potatoes (ground), peas (ground), animal fat, peas starch, brewer’s yeast, minerals, tomatoes (ground), carrots (ground), salmon oil, flax oil, cranberry, inulin (source for FOS), *yucca schidigera* powder	“Black soldier fly” in the composition
80	*H. illucens*	Treats for dogs	37	Germany	Carrot 75.77%, black soldier fly (*Hermetia illucens*) 23.83%, guar gum	“*Hermetia illucens*” in the composition
81	*H. illucens*	Pet food for turtles	38	Poland	Fish and fish derivatives, derivatives of vegetable origin, vegetable protein extracts, cereals, molluscs and crustaceans (*Gammarus pulex* 4.8%), algae, insects (meal from *Hermetia illucens* larvae 3%), yeasts, oils and fats, minerals	“*Hermetia illucens*” in the composition
82	*H. illucens*	Feed for ornemental fish	39	Germany	Insect protein (*Hermetia*) 15%, wheat flour, shrimp flour, wheat germ, rice flour, salmon flour, squid flour, soy flour, yeast extract	“*Hermetia*” in the composition
83	*H. illucens*	Feed for ornemental fish	40	Germany	Black soldier fly 25%, salmon 23%, fish protein concentrate, wheat, potato, shrimp meal, dicalcium phosphate, calcium carbonate, calendula, rosemary	“Black soldier fly” in the composition
84	*H. illucens*	Pet food for dogs	41	Germany	50% insects*, 35.5% broth, 5% potatoes, 5% carrots, 3% potato flakes, 0.5% herbs, 1% minerals. *Hermetia illucens larvae (black soldier fly)	“*Hermetia illucens*” in the composition
85	*H. illucens*	Treats for cats	42	France	Wheat, barley, maize, wheat bran, insect flour 11%, linseed cake, yellow pea protein, poultry fat, brewer’s yeast (min. 4%), animal protein hydrolysate	“Insect” in the composition and “Our kibble is made from *Hermetia Illucens* larvae” in the information mentioned on the site
86	*H. illucens*	Treats for cats	42	France	Wheat, barley, maize, wheat bran, insect flour 11%, linseed cake, yellow pea protein, poultry fat, dried dandelion leaves (min. 4%), animal protein hydrolysate	“Insect” in the composition and “Our kibble is made from *Hermetia Illucens* larvae” in the information mentioned on the site
87	*H. illucens*	Treats for dogs	43	Czech Republic	Insect protein 26%, peas, salmon protein 14%, potato starch, coconut oil, linseed 4%, dried apple pulp, hydrolyzed chicken liver, dried thyme 1%, dried algae (1%, *Schizochytrium limacinum*), rapeseed oil	“Insect” in the name of product and in the composition and “Black soldier fly larvae” on a reseller’s website
88	*H. illucens*	Treats for dogs	43	Czech Republic	Insect protein 26%, peas, rabbit protein 14%, potato starch, coconut oil, linseed 4%, dried apple pulp, hydrolyzed chicken liver, dried fennel 1%, dried algae (1%, *Schizochytrium limacinum*), rapeseed oil	“Insect” in the name of product and in the composition and “Black soldier fly larvae” on a reseller’s website
89	*H. illucens*	Treats for dogs	44	The Netherlands	Apple, black soldier fly, potato flour, flaxseed oil	“Black soldier fly” in the composition and in the name of product
90	*H. illucens*	Treats for dogs and cats	45	France	Apple, black soldier fly, potato flour, flaxseed oil	“Black soldier fly” in the composition
91	*T. molitor*	Pet food for cats	46	France	Dehydrated insect 18.7%, maize, wheat, dehydrated poultry 10.3%, peas 7.5%, wheat bran, poultry fat, hydrolyzed animal proteins, chicory fiber 0.9%, potassium chloride 0.5%, DL Methionine 0.5%, spirulina 0.2%, dehydrated nettle 0.2%, dehydrated gentian 0.1%	“Kibble with mealworms” indication on the packaging
92	*T. molitor*	Pet food for dogs	47	France	*Tenebrio molitor* 44.3%, sorghum, rapeseed oil 9%, mineral salts, linseed, hydrolyzed proteins, apple pomace, psyllium husk, brewer’s yeast 0.5%, fructo-oligo-saccharides, yeast cell walls (source of mannan-oligo-saccharides and beta-glucans), artichoke, glucosamine sulfate, chondroitin sulfate	“*Tenebrio molitor*” in the composition
93	*T. molitor*	Treats for dogs	4	France	Composition not provided	
94	*T. molitor*	Treats for dogs and cats	45	France	Pumpkin, mealworm, potato flour, flaxseed oil	“Mealworm” in the composition
95	*T. molitor*	Treats for dogs	44	The Netherlands	Pumpkin, mealworm, potato flour, flaxseed oil	“Mealworm” in the composition and in the name of product
96	*T. molitor*	Pet food for backyard hens and poultry	46	France	Powdered dehydrated mealworms, maize, wheat, barley, peas, faba beans, field beans	“Mealworms” in the name of product
97	*T. molitor*	Pet food for dogs	46	France	Dehydrated insect 21.4%, wheat, maize, wheat bran, peas 9.3%, dehydrated poultry 8.4%, poultry fat, hydrolyzed animal proteins, potassium chloride 0.5%, dehydrated nettle 0.2%, dehydrated gentian 0.05%, dehydrated thyme 0.05%	“Kibble with mealworms” indication on the packaging
98	*T. molitor*	Pet food for dogs	46	France	Dehydrated insect 20.7%, wheat, maize, dehydrated poultry 13.5%, peas 8%, wheat bran, poultry fat, hydrolyzed animal proteins, potassium chloride 0.5%, dehydrated nettle 0.2%, dehydrated thyme 0.1%, dehydrated hawthorn	“Kibble with mealworms” indication on the packaging
99	*T. molitor*	Pet food for cats	4	France	Dehydrated mealworms 20%, dehydrated poultry, wheat, corn, green Limagne lentil, wheat bran, poultry fat, hydrolyzed animal proteins, chicory fiber (1%, including fructo-oligosaccharides 0.6%), potassium chloride, dehydrated spirulina (200 mg/kg), dehydrated nettle (200 mg/kg), dehydrated gentian (200 mg/kg)	“Mealworms” in the composition
100	*T. molitor*	Feed for ornamental fish	48	Germany	*Tenebrio* larvae 40%, wheat gluten, wheat flour, wheat germ, brewer’s yeast, seaweed, rapeseed oil, Mannan oligosaccharides, green-lipped mussel, garlic, *Haematococcus algae*	“*Tenebrio molitor*” in the composition
101	*B. mori*	Feed for ornamental fish	49	Japan	Insects, cereals, by-products of plant origin, algae, mineral substances	“Silkworm” in the name of product
102	*B. mori*	Fishing bait	33	The Netherlands	Maize, wheat, silkworm meal, soy flour, chicken protein powder, egg powder, mulberry Florentine aroma, salt	“*Bombyx mori*” in the name of product
103	*B. mori*	Feed for ornamental fish	39	Germany	Salmon meal 23%, silkworms 14%, wheat germs, maize meal, krill meal 9%, gammares, soja meal, rice meal, wheat gluten, spirulina 4%, yeast	“Silkworm” in the name of product
104	*B. mori*	Treats for dogs and cats	45	France	Carrot, mulberry bombyx, potato flour, flaxseed oil	“Mulberry bombyx” in the name of product
105	*B. mori*	Treats for dogs	44	The Netherlands	Carrot, silkworm, potato flour, flaxseed oil	“Silkworm” in the name of product
106	Cricket	Treats for dogs	44	The Netherlands	Sweet potato, cricket, potato flour, flaxseed oil	“Cricket” in the composition and in the name of product
107	Cricket	Treats for dogs	45	France	Sweet potato, cricket, potato flour, flaxseed oil	“Cricket” in the composition and in the name of product
108	Unspecified	Fishing bait	50	France	Insect meal	“Insect” in the name of product
109	Unspecified	Pet food for dogs	51	The Netherlands	Potato, insect meal, poultry fat, vitamins and minerals, yeast (hydrolysate), calcium carbonate, fish oil, linseed	“Insects” in the composition and in the name of product
110	Unspecified	Pet food for cats	52	Switzerland	Dried whole eggs 21%, dried potatoes, potato starch, pea protein, insect protein meal 7%, animal fat, minerals, vegetable oil, digest, dried beet pulp, flaxseed, fish oil, dried carrots, dried tomato pomace, dried citrus pulp, spinach powder	“Insects” in the composition
111	Unspecified	Fishing bait	34	The Netherlands	No information	“Insect” in the name of product
112	*H. illucens, B. mori*, insect powder	Fishing bait	34	The Netherlands	Animal meals, vegetable meals, enhancers, amino acids, preserver, flavor, sweetener, vitamins, minerals	“Crafted with several insect meals such as black soldier fly meal, silkworm and insect powder” in the information mentioned on the site
113	*H. illucens, B. mori*	Fishing bait	34	The Netherlands	Animal meals, vegetable meals, enhancers, amino acids, preserver, flavor, sweetener, vitamins, minerals	“Crafted with defatted black soldier fly meal and silkworm meal” in the information mentioned on the site
114	*H. illucens*, *T. molitor*, *B. mori*	Pet food for reptile	38	Poland	Freeze-dried fruits 40% (strawberry 15%, banana, pineapple), insects (mealworm meal 13%, silkworm pupa meal 7%, *Hermetia illucens* larvae meal 5%), bee products 12% (honey powder, ollen), whey protein powder, derivatives of plant origin, micellar casein, dried marigold flowers, seeds (linseed), minerals (calcium carbonate), algae, magnesium stearate, yeast, calcium citrate, calcium gluconate	“Mealworm”, “silkworm” and “*Hermetia illucens*” in the composition
115	*T. molitor*, *Gryllus testaceus*	Pet food for dogs	53	Germany	28.7% carrots, 27% peanut butter protein, 20% dried mealworms (*Tenebrio molitor*), tapioca starch, 5% dried crickets (*Gryllus testaceus*)	“*Tenebrio molitor*” and “*Gryllus testaceus*” in the composition
116	*T. molitor*, *A. domesticus*	Feed for ornamental fish	38	Poland	Molluscs and crustaceans (including dried shrimps 20%), fish and fish derivatives, cereals, derivatives of vegetable origin, vegetable protein extracts, insects (dried mealworms 8%, dried crickets 2%), algae (including Spirulina platensis 2.2%), yeasts, oils and fats, minerals	“Mealworms” and “crickets” in the composition
117	*H. illucens*, *T. molitor*, *B. mori*	Feed for ornamental fish	38	Poland	Insects (*Hermetia illucens* larvae meal 15%, silkworm pupae meal 15%, mealworms meal 15%), cereals, vegetable protein extracts, algae, oils, fats, minerals	“*Hermetia illucens*, silkworm, mealworms” in the composition
118	*H. illucens*, *T. molitor*	Feed for ornamental fish	40	Czech Republic	Insect meal (mealworm meal 15%, black soldier fly larvae 10%) wheat flour, wheat gluten, wheat germ, alfalfa, spirulina, fish protein hydrolyzed, kelp 5%, shrimp protein hydrolyzed, spinach 5%, activated charcoal	“Mealworm” and “black soldier fly” in the composition
119	*H. illucens*, *T. molitor*, *B. mori*, cricket	Pet food for reptile	54	United Kingdom	Black soldier fly larvae, papaya, calcium carbonate, coconut milk, locust bean gum, silkworm, banana, mealworm, apple, cricket, raspberry, honey, blueberry, vitamin B, bee pollen	“Black soldier fly larvae, silkworm, mealworm, cricket” in the composition

**Table 5:** Primers and probes from literature used to authenticate insects present in industrial samples labeled as containing insects.

Target	Name	Sequences 5′-3′	Publication
*Tenebrio molitor*	Cadherin-2F	AATAGACGAAGACAACCAGCTTGA	[47]
	Cadherin-2R	TCTCTATCGGCATCACTATATGTTAGATT	
	Cadherin-2P	FAM–CCGGACGACACCCTCAACGGA–TAMRA	
*Hermetia illucens*	HI-mito-2F	ACCATTCTTCAAGCCTATGA	[44]
	HI-mito-2R	TTGAGCCGTAGACTGCG	
	HI-mito-P	FAM–TGAAGCCCCTTTTACTATTGCTG–TAMRA	
*Alphitobius diaperinus*	Alphi-Dia-Cad-F	CCAAGTGACTCTCATCATTCAGGAT	[46]
	Alphi-Dia-Cad-R	CTGAAACCGTAATGTCTAGTTCACCTA	
	Alphi-Dia-Cad-P	FAM–CCATTGCAGATCCAAGTCCCCGAAA–TAMRA	
*Acheta domesticus*	AchetaD_cytB_F1	ATAGTAGGTATTCTAATCTTATTCCTA	[39]
	AchetaD_cytB_R1	CATTGTACTAGATCAGTTCCTAGATA	
	AchetaD_cytB_P1	FAM–AATAGCTGCCGCTTTCATAGGTTAC–TAMRA	
*Bombyx* *mori*	Bombyx-Cad-F	TTTCAGACACCGACCATGACA	[45]
	Bombyx-Cad-R	CCAAAATGATGCCGAAGTACTG	
	Bombyx-Cad-P	FAM–AGCTCTGGAGCATTGTCGTTCACATCAA–TAMRA	
*Gryllodes sigillatus*	GS1Fw	GATCAAACAATCCCCTAGGTGTC	Ref. [59] with a modification as described in [39]
	GS1re	CTGGGTCTCCAAGTATATAAGGATTAG	

**Table 6:** Samples collected from industries or purchased online, categorized by insect species present in the sample and by the intended use of the product (food or feed). A total of 119 distinct samples were analyzed, of which 8 contained more than one insect species.

Sample Types		Samples Labeled to Contain									
		*T. molitor*	*H. illucens*	*A. diaperinus*	*A. domesticus*	*B. mori*	*G. sigillatus*	*G. assimilis*	*L. migratoria*	Crickets	Others or Not Specified
Food	Pure flour	10	0 *	4	7	0 *	2 *	0 *	1	4	0 °
	Multi-ingredient food	7	0 *	6	11	0 *	0 *	0 *	0 °	3	0 °
Feed	Pure meal	5	9	0 °	0 °	2	0 °	2	0 *	1	3
	Multi-ingredient feed	16	20	0 °	1	10	0 °	0 °	0 *	3	4
	Total	38	29	10	19	12	2	2	1	11	7

* = not authorized in the European Union; ° = not found as well for completeness.

**Table 7:** Results obtained by real-time PCR tests and high-throughput sequencing (HTS, i.e., metabarcoding) for 28 single-species insect flour samples. PCR results are based on undiluted extracts. For positive results, mean Cq values obtained from 2 DNA extracts are given in brackets. Expected PCR positive results are marked with a gray background. Possible expected results when the species is not indicated are marked with a blue background. (Real-time PCR settings on LightCycler 480 were set to second derivative max and high confidence). HTS results represent the relative read abundance (%) of each species detected based on *COI*, *12S*, and *28S* metabarcoding. Percentages indicate the proportion of total DNA barcode reads assigned to each species per sample, averaged across the results provided by the three metabarcoding markers.

		Results with PCR Tests for the Detection of						
Sample No.	Expected Species	*T. molitor*	*H. illucens*	*A. diaperinus*	*B. mori*	*A. domesticus*	*G. sigillatus*	HTS
1	*T. molitor*	+(27)	-	-	-	-	-	*T. molitor* (100%)
2	*T. molitor*	+(27)	-	-	-	-	-	*T. molitor* (100%)
3	*T. molitor*	+(25)	-	-	-	-	-	*T. molitor* (100%)
4	*T. molitor*	+(24)	-	-	-	-	-	*T. molitor* (100%)
5	*T. molitor*	+(21)	-	-	-	-	-	*T. molitor* (100%)
6	*T. molitor*	+(27)	+(32)	+(35)	-	-	-	*T. molitor* (100%)
7	*T. molitor*	+(25)	-	+(36)	-	+(38)	-	*T. molitor* (100%)
8	*T. molitor*	+(26)	-	-	-	+(36)	-	*T. molitor* (100%)
9	*T. molitor*	+(24)	-	-	-	+(38)	-	*T. molitor* (100%)
10	*T. molitor*	+(24)	-	-	-	+(39)	-	*T. molitor* (100%)
11	*A. domesticus*	-	-	-	-	+(18)	-	*A. domesticus* (100%)
12	*A. domesticus*	-	-	-	-	+(16)	-	*A. domesticus* (99.1%), *Dermestes ater* (0.9%)
13	*A. domesticus*	-	-	-	-	+(18)	-	*A. domesticus* (100%)
14	*A. domesticus*	+(38)	-	-	-	+(18)	-	*A. domesticus* (100%)
15	*A. domesticus*	+(38)	-	-	-	+(16)	-	*A. domesticus* (100%)
16	*A. domesticus*	+(37)	+(33)	-	-	+(15)	-	*A. domesticus* (100%)
17	*A. domesticus*	-	+(34)	-	-	+(18)	+(39)	*A. domesticus* (44.7%), *G. assimilis* (55.3%)
18	*A. diaperinus*	-	-	+(26)	-	-	-	*A. diaperinus* (100%)
19	*A. diaperinus*	-	-	+(24)	-	-	-	*A. diaperinus* (100%)
20	*A. diaperinus*	+(30)	-	+(21)	-	+(31)	-	*A. diaperinus* (97.4%), *T. molitor* (2.6%)
21	*A. diaperinus*	-	+(38)	+(20)	-	-	-	*A. diaperinus* (100%)
22	*G. sigillatus*	-	-	+(39)	-	+(36)	+(19)	*G. sigillatus* (100%)
23	*G. sigillatus*	-	-	-	-	+(22)	-	*A. domesticus* (100%)
24	*L. migratoria*	+(36)	-	+(36)	-	+(31)	-	*L. migratoria* (100%)
25	Cricket	-	-	-	-	+(15)	-	*A. domesticus* (100%)
26	Cricket	-	-	-	-	+(19)	-	*A. domesticus* (88.8%), *G. assimilis* (11.2%)
27	Cricket	-	-	-	-	+(19)	-	*A. domesticus* (85.5%), *G. assimilis* (14.5%)
28	Cricket	-	-	-	-	+(18)	+(39)	*A. domesticus* (87.3%), *G. assimilis* (12.7%)

**Table 8:** Results obtained by official real-time PCR tests for the detection of animal proteins in 28 single-species insect flour samples. PCR results are based on undiluted extracts. For positive results, mean Cq values obtained from 2 DNA extracts are given in brackets. (Real-time PCR settings on LightCycler 480 set to second derivative max and high confidence for ruminant and poultry targets and fit points for porcine).

		Results with PCR Tests for the Detection of		
Sample No.	Expected Species	Ruminant (Cut-Off Value at 36.6 Cycles)	Porcine (Cut-Off Value at 39.2 Cycles)	Poultry (Cut-Off Value at 37.7 Cycles)
1	*T. molitor*	-	-	-
2	*T. molitor*	-	-	-
3	*T. molitor*	-	-	-
4	*T. molitor*	-	-	-
5	*T. molitor*	-	-	-
6	*T. molitor*	-	-	-
7	*T. molitor*	-	-	-
8	*T. molitor*	-	-	-
9	*T. molitor*	-	-	-
10	*T. molitor*	-	-	-
11	*A. domesticus*	+(35)	-	-
12	*A. domesticus*	+(33)	-	-
13	*A. domesticus*	+(32)	+(35)	-
14	*A. domesticus*	-	-	-
15	*A. domesticus*	+(36)	-	-
16	*A. domesticus*	-	-	-
17	*A. domesticus*	+(29)	-	-
18	*A. diaperinus*	-	-	-
19	*A. diaperinus*	-	-	-
20	*A. diaperinus*	-	-	-
21	*A. diaperinus*	-	-	-
22	*G. sigillatus*	-	-	-
23	*G. sigillatus*	-	-	-
24	*L. migratoria*	-	-	-
25	Cricket	-	-	-
26	Cricket	+(32)	-	-
27	Cricket	+(31)	-	-
28	Cricket	+(31)	-	-

**Table 9:** Results obtained by real-time PCR tests and high-throughput sequencing (HTS, i.e., metabarcoding) for 27 multi-ingredient food labeled to contain insect. PCR results are based on undiluted extracts. For positive results, mean Cq values obtained from 2 DNA extracts are given in brackets. Expected PCR positive results are marked with a gray background. Possible expected results when the species is not indicated are marked with a blue background. (Real-time PCR settings on LightCycler 480 were set to second derivative max and high confidence). HTS results represent the relative read abundance (%) of each species detected based on *COI*, *12S*, and *28S* metabarcoding. Percentages indicate the proportion of total DNA barcode reads assigned to each species per sample, averaged across the results provided by the three metabarcoding markers.

		Results with PCR Tests for the Detection of						
Sample No.	Expected Species	*T. molitor*	*H. illucens*	*A. diaperinus*	*B. mori*	*A. domesticus*	*G. sigillatus*	HTS
29	*T. molitor*	+(28)	-	-	-	-	-	*T. molitor* (100%)
30	*T. molitor*	+(28)	-	-	-	-	-	*T. molitor* (100%)
31	*T. molitor*	+(31)	-	-	-	-	-	*T. molitor* (100%)
32	*T. molitor*	+(26)	-	-	-	-	-	*T. molitor* (100%)
33	*T. molitor*	+(29)	-	-	-	-	-	*T. molitor* (100%)
34	*T. molitor*	+(24)	-	-	-	+(27)	-	*T. molitor* (100%)
35	*T. molitor*	+(29)	+(35)	-	-	-	-	*T. molitor* (100%)
36	*A. domesticus*	-	-	-	-	+(23)	-	*A. domesticus* (100%)
37	*A. domesticus*	-	-	-	-	+(25)	-	*A. domesticus* (100%)
38	*A. domesticus*	-	-	-	-	+(24)	-	*A. domesticus* (100%)
39	*A. domesticus*	-	-	-	-	+(26)	-	*A. domesticus* (100%)
40	*A. domesticus*	-	-	-	-	+(24)	-	*A. domesticus* (100%)
41	*A. domesticus*	-	-	-	-	+(24)	-	*A. domesticus* (99.8%), *T. molitor* (0.2%)
42	*A. domesticus*	-	-	-	-	+(21)	-	*A. domesticus* (100%)
43	*A. domesticus*	-	-	-	-	+(22)	-	*A. domesticus* (68.2%), *G. assimilis* (31.8%)
44	*A. domesticus*	-	-	-	-	+(26)	-	*A. domesticus* (86.2%), *G. assimilis* (13.8%)
45	*A. domesticus*	-	-	-	-	+(23)	-	*A. domesticus* (94.0%), *G. bimaculatus* (4.5%), *G. assimilis* (1.5%)
46	*A. domesticus*	-	-	-	-	+(17)	-	*A. domesticus* (100%)
47	*A. diaperinus*	-	-	+(29)	-	-	-	*A. diaperinus* (100%)
48	*A. diaperinus*	-	-	+(24)	-	+(29)	-	*A. diaperinus* (100%)
49	*A. diaperinus*	-	-	+(30)	-	+(35)	-	*A. diaperinus* (100%)
50	*A. diaperinus*	-	-	+(28)	-	+(34)	-	*A. diaperinus* (100%)
51	*A. diaperinus*	-	-	+(29)	-	+(33)	-	*A. diaperinus* (97.4%), *Callosobruchus maculatus* (2.6%)
52	*A. diaperinus*	-	-	-	-	-	-	*A. diaperinus* (100%)
53	Cricket	+(35)	-	-	-	+(22)	-	*A. domesticus* (99.6%), *T. molitor* (0.4%)
54	Cricket	-	-	-	-	+(25)	-	*A. domesticus* (100%)
55	Cricket	-	-	-	-	+(26)	-	*A. domesticus* (87.0%), *G. assimilis* (13.0%)

**Table 10:** Results obtained by real-time PCR tests and high-throughput sequencing (HTS, i.e., metabarcoding) for 21 single-species insect meal samples. PCR results are based on undiluted extracts, except when marked with a letter referring to a dilution used. For positive results, mean Cq values obtained from 2 DNA extracts are given in brackets. Expected PCR positive results are marked with a gray background. Potentially expected results in cases where the species is not indicated are marked with a blue background. (Real-time PCR settings on LightCycler 480 were set to second derivative max and high confidence). HTS results represent the relative read abundance (%) of each species detected based on *COI*, *12S*, and *28S* metabarcoding. Percentages indicate the proportion of total DNA barcode reads assigned to each species per sample, averaged across the results provided by the three metabarcoding markers.

		Results with PCR Tests for the Detection of						
Sample No.	Expected Species	*T. molitor*	*H.* *illucens*	*A. diaperinus*	*B. mori*	*A. domesticus*	*G. sigillatus*	HTS
56	*H. illucens*	-	+(15)	-	-	-	-	*H. illucens* (100%)
57	*H. illucens*	-	+(16)	-	-	-	-	*H. illucens* (100%)
58	*H. illucens*	-	+(16)	-	-	-	-	*H. illucens* (100%)
59	*H. illucens*		+(18)	-	-	-	-	*H. illucens* (100%)
60	*H. illucens*	-	+(16)	-	-	-	-	*H. illucens* (100%)
61	*H. illucens*	-	+(16)	-	-	-	-	*H. illucens* (100%)
62	*H. illucens*	-	+(20)	-	-	-	-	*H. illucens* (100%)
63	*H. illucens*	-	+(21) ^a^	-	-	-	-	*H. illucens* (100%)
64	*H. illucens*	+(37)	+(22) ^a^	-	-	-	-	*H. illucens* (78.1%), *Gromphadorhina portentosa* (21.9%)
65	*T. molitor*	+(26)	-	-	-	-	-	*T. molitor* (100%)
66	*T. molitor*	+(27)	-	-	-	-	-	*T. molitor* (100%)
67	*T. molitor*	+(37)	+(37)	-	-	-	-	*T. molitor* (98.6%), *Sitophilus zeamais* (1.4%)
68	*T. molitor*	+(28)	+(36)	+(33)	-	-	-	*T. molitor* (94.4%), *A. diaperinus* (5.6%)
69	*T. molitor*	+(22)	-	-	-	+(38) ^a^	-	*T. molitor* (100%)
70	*B. mori*	-	+(31)	-	+(23)	-	-	*B. mori* (71.1%), *Tribolium castaneum* (16.2%), *Necrobia rufipes* (12.7%)
71	*B. mori*	-	+(34)	-	+(23)	-	-	*B. mori* (95.7%), *Chrysomya megacephala* (3.9%), *M. domestica* (0.3%),
72	Cricket	-	-	+(35)	-	-	+(21)	*G. sigillatus* (100%)
73	*G. assimilis*	-	-	-	-	-	-	*G. assimilis* (100%)
74	*G. assimilis*	-	+(34)	+(39)	-	-	-	*G. assimilis* (100%)
75	*Blabtica dubia*	+(31)	+(33) ^a^	-	-	-	-	*Blabtica dubia* (81.3%), *T. molitor* (18.7%)
76	*Gromphadorhina portentosa*	+(35)	-	+(39)	-	-	-	*Gromphadorhina portentosa* (100%)

^a^ = 10-fold dilution of extracts DNA.

**Table 11:** Results obtained by official real-time PCR tests for the detection of animal proteins in 21 single-species insect meal samples. PCR results are based on undiluted extracts, except when marked with a letter referring to a dilution used. For positive results, mean Cq values obtained from 2 DNA extracts are given in brackets. (Real-time PCR settings on LightCycler 480 were set to second derivative max and high confidence for ruminant and poultry and fit points for porcine).

		Results with PCR Tests for the Detection of		
Sample No.	Expected Species	Ruminant (Cut-Off Value at 36.6 Cycles)	Porcine (Cut-Off Value at 39.2 Cycles)	Poultry (Cut-Off Value at 37.7 Cycles)
56	*H. illucens*	-	-	-
57	*H. illucens*	-	-	-
58	*H. illucens*	-	-	-
59	*H. illucens*	-	-	-
60	*H. illucens*	-	-	-
61	*H. illucens*	+(34)	-	-
62	*H. illucens*	+(32)	-	+(25)
63	*H. illucens*	-	-	+(19) ^a^
64	*H. illucens*	-	-	-
65	*T. molitor*	-	-	-
66	*T. molitor*	-	-	-
67	*T. molitor*	+(30)	+(31)	+(35)
68	*T. molitor*	-	-	-
69	*T. molitor*	-	-	-
70	*B. mori*	+(36)	-	-
71	*B. mori*	-	-	-
72	Cricket	+(33)	-	-
73	*G. assimilis*	-	-	-
74	*G. assimilis*	-	-	-
75	*Blabtica dubia*	-	-	-
76	*Gromphadorhina portentosa*	-	-	-

^a^ = 10 fold dilution of extracts DNA.

**Table 13:** Results obtained by real-time PCR tests and high-throughput sequencing (HTS, i.e., metabarcoding) on samples from two different brands (brands 44 and 45) labeled as containing a single species. PCR results are based on undiluted extracts. For positive results, mean Cq values obtained from 2 DNA extracts are given in brackets. Expected PCR positive results are marked with a gray background. Potentially expected results in cases where the species is not indicated are marked with a blue background. (Real-time PCR settings on LightCycler 480 were set to second derivative max and high confidence). HTS results represent the relative read abundance (%) of each species detected based on *COI*, *12S*, and *28S* metabarcoding. Percentages indicate the proportion of total DNA barcode reads assigned to each species per sample, averaged across the results provided by the three metabarcoding markers.

			Results with PCR Tests for the Detection of						
Brand No.	Sample No.	Expected Species	*T. molitor*	*H. illucens*	*A. diaperinus*	*B. mori*	*A. domesticus*	*G. sigillatus*	HTS
44	89	*H. illucens*	+(30)	+(34)	-	-	-	-	*T. molitor* (100%)
	95	*T. molitor*	+(30)	+(32)	-	-	-	-	*T. molitor* (100%)
	105	*B. mori*	+(30)	+(36)	-	-	-	-	*T. molitor* (100%),
	106	Cricket	+(31)	-	-	-	-	-	*T. molitor* (100%)
45	90	*H. illucens*	+(32)	-	-	-	-	-	*T. molitor* (100%)
	94	*T. molitor*	+(31)	-	-	-	-	-	*T. molitor* (100%)
	104	*B. mori*	+(33)	+(23)	-	-	-	-	*T. molitor* (77.8%), *H. illucens* (21.9%), *Sitophilus oryzae* (0.2%)
	107	Cricket	+(34)	-	-	-	-	-	*T. molitor* (100%)

**Table 14:** Results obtained by real-time PCR tests and high-throughput sequencing (HTS, i.e., metabarcoding) on 4 multi-ingredient feed samples for which the insect species was unspecified. PCR results are based on undiluted extracts, except when marked with a letter referring to a dilution used. For positive results, mean Cq values obtained from 2 DNA extracts are given in brackets.(Real-time PCR settings on LightCycler 480 were set to second derivative max and high confidence). HTS results represent the relative read abundance (%) of each species detected based on *COI*, *12S*, and *28S* metabarcoding. Percentages indicate the proportion of total DNA barcode reads assigned to each species per sample, averaged across the results provided by the three metabarcoding markers.

		Results with PCR Tests for the Detection of						
Sample No.	Expected Species	*T. molitor*	*H. illucens*	*A. diaperinus*	*B. mori*	*A. domesticus*	*G. sigillatus*	HTS
108	Unspecified	-	+(16)	-	-	-	-	*H. illucens* (100%)
109	Unspecified	-	+(19)	-	-	-	-	*H. illucens* (100%)
110	Unspecified	-	+(25) ^a^	-	-	-	-	*H. illucens* (100%)
111	Unspecified	-	+(18)	-	-	-	-	*H. illucens* (100%)

^a^ = 10-fold dilution of extracts DNA.

**Table 15:** Results obtained by real-time PCR tests and high-throughput sequencing (HTS, i.e., metabarcoding) on 8 multi-ingredient feed samples labeled as containing multiple insect species. PCR results are based on undiluted extracts, except when marked with a letter referring to a dilution used. For positive results, mean Cq values obtained from 2 DNA extracts are given in brackets. Expected PCR positive results are marked with a gray background. Potentially expected results in cases where the species is not indicated are marked with a blue background. (Real-time PCR settings on LightCycler 480 were set to second derivative max and high confidence). HTS results represent the relative read abundance (%) of each species detected based on *COI*, *12S*, and *28S* metabarcoding. Percentages indicate the proportion of total DNA barcode reads assigned to each species per sample, averaged across the results provided by the three metabarcoding markers.

		Results with PCR Tests for the Detection of						
Sample No.	Expected Species	*T. molitor*	*H. illucens*	*A. diaperinus*	*B. mori*	*A. domesticus*	*G. sigillatus*	HTS
112	*H. illucens, B. mori*, insect powder	-	+(22)	-	+(30)	-	-	*B. mori* (1.1%), *H. illucens* (98.9%)
113	*H. illucens, B. mori*	-	+(18)	-	+(29)	-	-	*H. illucens* (99.6%), *B. mori* (0.4%)
114	*H. illucens*, *T. molitor*, *B. mori*	+(39)	+(28) ^a^	-	+(33) ^a^	-	-	*B. mori* (93.5%), *H. illucens* (6.5%)
115	*T. molitor*, *G. testaceus*	+(31)	+(36)	-	-	-	-	*T. molitor* (74.2%), *Teleogryllus emma* (20.2%), *Loxoblemmus doenitzi* (3.6%), *Loxoblemmus equestris* (2.0%)
116	*T. molitor*, *A. domesticus*	+(32) ^a^	+(32) ^b^	-	-	+(34) ^a^	-	*T. molitor* (92.7%), *A. domesticus* (1.1%), *Teleogryllus emma* (6.2%)
117	*H. illucens*, *T. molitor*, *B. mori*	-	+(25) ^c^	-	+(30) ^b^	-	-	*B. mori (16.1%)*, *H. illucens (70.3%)*, *Chrysomya megacephala* (13.6%)
118	*H. illucens*, *T. molitor*	-	+(27) ^d^	-	-	-	-	*H. illucens* (98.5%), *Daphnia pulex* (1.5%)
119	*H. illucens*, *T. molitor*, *B. mori*, cricket	-	+(17)	-	-	-	-	*H. illucens* (100%)

^a^ = 10-fold dilution, ^b^ = 20-fold dilution, ^c^ = 40-fold dilution, ^d^ = 160-fold dilution of extracts DNA.

## Data Availability

The reference databases used in this work (*COI*, *12S*- and *28S rRNA* genes), together with databases dedicated to other loci (*COII*, *CytB*, *16S* and *18S rRNA* genes), are freely available (https://doi.org/10.6084/m9.figshare.29313773). The TDmerger script, used to infer consensus taxonomic composition, is available on GitHub (https://github.com/benn888/TDmerger, accessed on 8 July 2025).
